# Green armoured tardigrades (Echiniscidae: *Viridiscus*), including a new species from the Southern Nearctic, exemplify problems with tardigrade variability research

**DOI:** 10.1038/s41598-023-40609-4

**Published:** 2023-09-28

**Authors:** Sogol Momeni, Piotr Gąsiorek, Jacob Loeffelholz, Stanislava Chtarbanova, Diane R. Nelson, Rebecca Adkins Fletcher, Łukasz Michalczyk, Jason Pienaar

**Affiliations:** 1 Department of Biological Sciences, University of Alabama, Tuscaloosa, AL USA; 2 Department of Invertebrate Evolution, Institute of Zoology and Biomedical Research, Faculty of Biology, Jagiellonian University, Kraków, Poland; 3 Natural History Museum of Denmark, University of Copenhagen, Copenhagen, Denmark; 4 Department of Biological Sciences, East Tennessee State University, Johnson City, TN USA; 5 Department of Appalachian Studies, East Tennessee State University, Johnson City, TN USA; 6 Department of Biological Sciences and the Institute of Environment, Florida International University, Miami, FL USA

**Keywords:** Biodiversity, Biogeography, Phylogenetics, Taxonomy

## Abstract

Ranges of tardigrade intraspecific and interspecific variability are not precisely defined, both in terms of morphology and genetics, rendering descriptions of new taxa a cumbersome task. This contribution enhances the morphological and molecular dataset available for the heterotardigrade genus *Viridiscus* by supplying new information on Southern Nearctic populations of *V.* *perviridis*, *V.* *viridianus*, and a new species from Tennessee. We demonstrate that, putting aside already well-documented cases of significant variability in chaetotaxy, the dorsal plate sculpturing and other useful diagnostic characters, such as morphology of clavae and pedal platelets, may also be more phenotypically plastic characters at the species level than previously assumed. As a result of our integrative analyses, *V.* *viridianus* is redescribed, *V.* *celatus* sp. nov. described, and *V.* *clavispinosus* designated as *nomen* *inquirendum*, and its junior synonymy with regard to *V.* *viridianus* suggested. Morphs of three *Viridiscus* species (*V.* *perviridis*, *V.* *viridianus*, and *V.* *viridissimus*) are depicted, and the implications for general echiniscid taxonomy are drawn. We emphasise that taxonomic conclusions reached solely through morphological or molecular analyses lead to a distorted view on tardigrade α-diversity.

## Introduction

Tardigrades, also known as water bears or moss piglets, represent an invertebrate phylum closely related to onychophorans and arthropods within Panarthropoda^1^, which consists of *ca*. 1500 described species to date^2^. These are grouped into two classes: Heterotardigrada and Eutardigrada. Heterotardigrades are characterised by the presence of cirrus *A*^3–5^, whereas eutardigrades are generally distinguishable by their simplified, vermiform external morphology^6^. Tardigrades inhabit limno-terrestrial and aquatic, both freshwater and marine, habitats, the former typically in association with bryophytes, algae, lichens, or leaf litter^5,7,8^. Although the number of documented tardigrade species has increased significantly in the past three decades, most regions globally remain sparsely sampled for these diminutive animals^9,10^. The distributions of a few terrestrial tardigrade species are well documented for some regions of North America, e.g. see^11^, but the general paucity of North American tardigrade specialists has left much of the continent unexplored in this regard (most recently reviewed in^12,13^).

The Echiniscidae, the most speciose family within the class Heterotardigrada^14^, contain the morphologically odd genus *Viridiscus*^15–17^. The former *Echiniscus* *viridis* group was established by Ramazzotti^18^, who considered *E.* *viridis* Murray, 1910^19^, *E.* *perviridis* Ramazzotti, 1959^18^, *E.* *viridissimus* Péterfi, 1956^20^, and *E.* *rufoviridis* du Bois-Reymond Marcus, 1944^21^ to be closely related based on green cuticular pigments in plates forming the armour, alongside orange pigments typically present in the body cavity of echiniscids^22^. Two further species were added to this group many decades later: *E.* *viridianus* Pilato et al., 2007^23^ and *E.* *clavispinosus* Fontoura et al., 2011^24^. *Viridiscus* was erected based on morphological and molecular data to accommodate the distinctiveness of this group: dark body pigments, well-developed, sabre-like claws, a lack of trunk appendages except cirrus *A*, and typical dorsal plate sculpture composed of an intracuticular sponge layer and small, flat, densely arranged epicuticular granules^15^. Recently, new evidence was presented, pointing out possibly large intraspecific variability within *Viridiscus*, as two morphs were distinguished within *V.* *viridissimus*^16,17^. Moreover, *V.* *rufoviridis* was transferred to *Barbaria*^25^, rectifying the earlier assignment of this species within the *viridis* group^18,23,26^. Consequently, *Viridiscus* currently comprises five species: *V.* *clavispinosus*, *V.* *perviridis*, *V.* *viridianus*, *V.* *viridis*, and *V.* *viridissimus*.

Here, we describe a new species of *Viridiscus* found in previously described *Viridiscus* assemblages in Tennessee^16,27^ and identified as *V*. aff. *viridianus* in^17^. We also redescribe *V.* *viridianus* based on multiple populations collected in Alabama and Florida. Newly obtained data confirm and strengthen previous findings that *Viridiscus* species are variable in the dorsal plate sculpturing^17^ and morphology of the primary clavae. We update the genus phylogeny, and question the validity of *V.* *clavispinosus*, which we hypothesise to be a synonym of *V.* *viridianus*. We stress that there is no convincing evidence for the presence of *V.* *viridis* outside of the Hawaiian Archipelago^26^, and the Nearctic records^28^ of this species are unreliable. The case of *Viridiscus* variability yet again illustrates that forming taxonomic conclusions based solely on morphology is unadvised and should be renounced^17^, especially since DNA sequence-based tools are readily available to incorporate into the systematics of these organisms.

## Results

### General remarks: morphology and phylogeny

**Figure 1:**
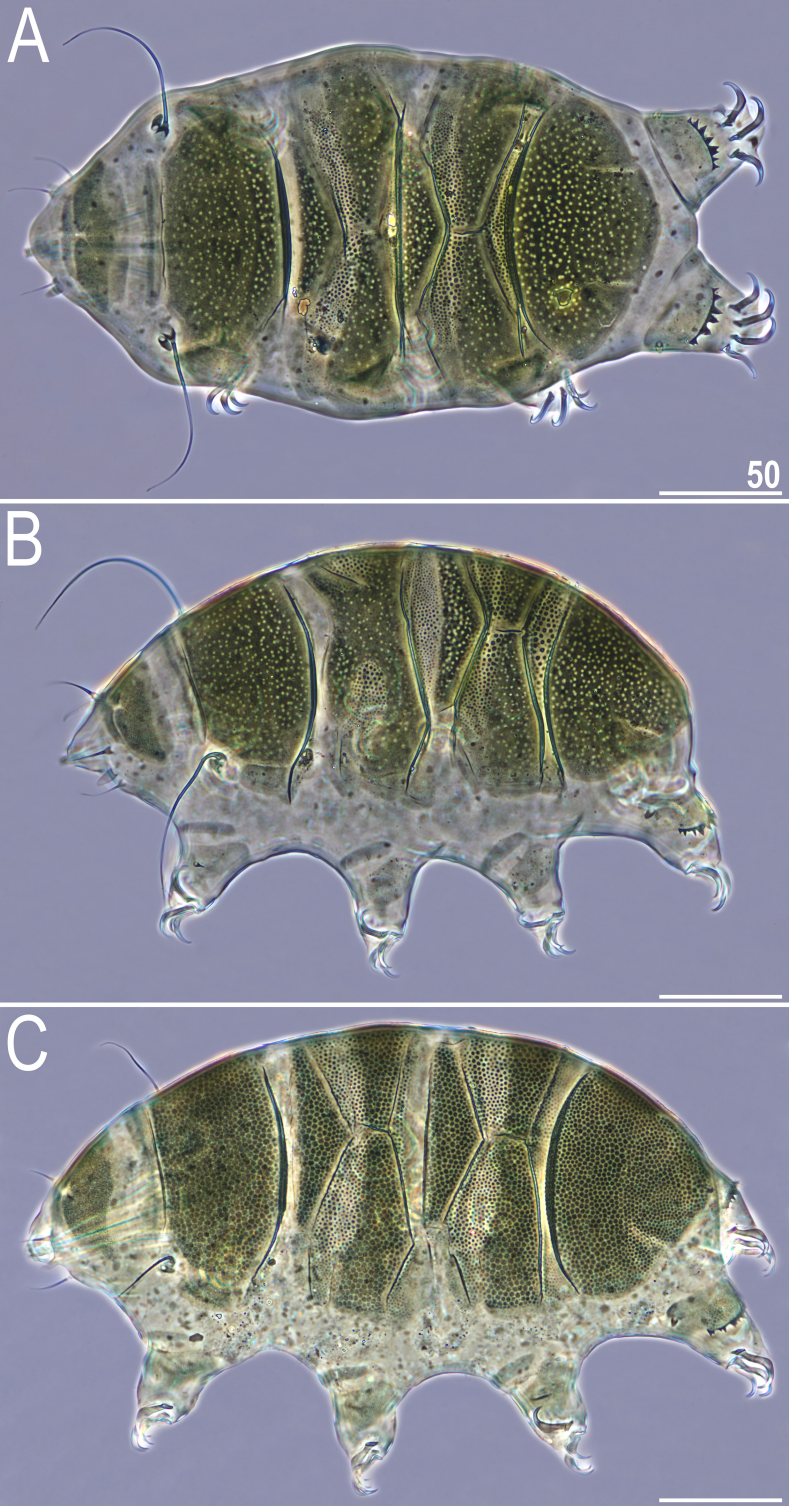
Two main morphotypes present in *Viridiscus* (PCM): (**A**) pores present, epicuticular granules typically reduced: the usual morph of *V.* *viridissimus* (Tennessee, dorsal view); (**B**) an aberrant specimen of *V.* *viridissimus* (Tennessee, dorsolateral view, the first median plate merged with the first paired segmental plate); (**C**) epicuticular granules dominant, pores present only in larval stage: *V.* *viridianus* (Alabama, dorsolateral view). Scale bars = 50 μm.

**Figure 2:**
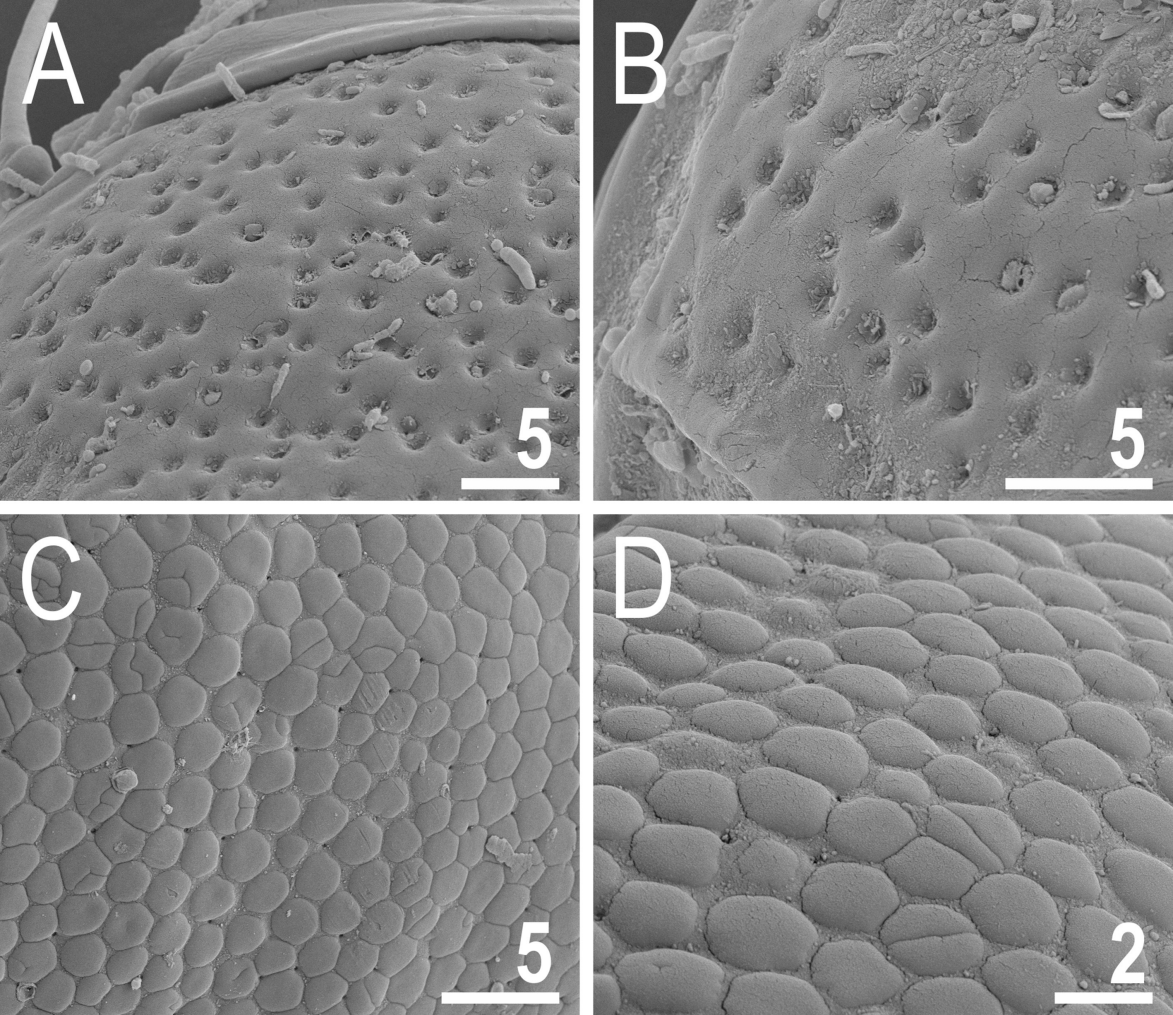
Two main morphotypes of dorsal plate sculpturing present in *Viridiscus* (SEM): pores dominant, only *V.* *viridissimus* (the population from Vietnam): (**A**) a fragment of the scapular plate; (**B**) close up of the posterior portion of the second paired segmental plate; epicuticular granules dominant, micropores visible only in SEM, all remaining *Viridiscus* species (the population of *V.* *perviridis* from Vietnam shown): (**C**) a fragment of the scapular plate; (**D**) close up of the central portion of the scapular plate. Scale bars in μm.

There are two general morphotypes distinguishable within *Viridiscus* with respect to the dorsal plate sculpturing: (I) pore-dominated, in which epicuticular granules are restricted to anterior portions of paired segmental plates (a usual morph of *V.* *viridissimus*, see Fig. 1A; however, we found several specimens with developmental aberrations embracing fusion of different elements of dorsal armour, see Fig. 1B); and (II) granule-dominated, in which pores are absent (the remaining four species, represented herein by *V.* *viridianus*: Fig. 1C). This division is also clearly visible in SEM: pores of *V.* *viridissimus* (Fig. 2A,B) are approaching the size of epicuticular granules of *V.* *perviridis* (Fig. 2C,D). However, the SEM analysis revealed also another element of sculpturing in the second *Viridiscus* morphotype: micropores, ≪ 1 μm in diameter and distributed irregularly between the granules (Fig. 2C,D).

**Figure 3:**
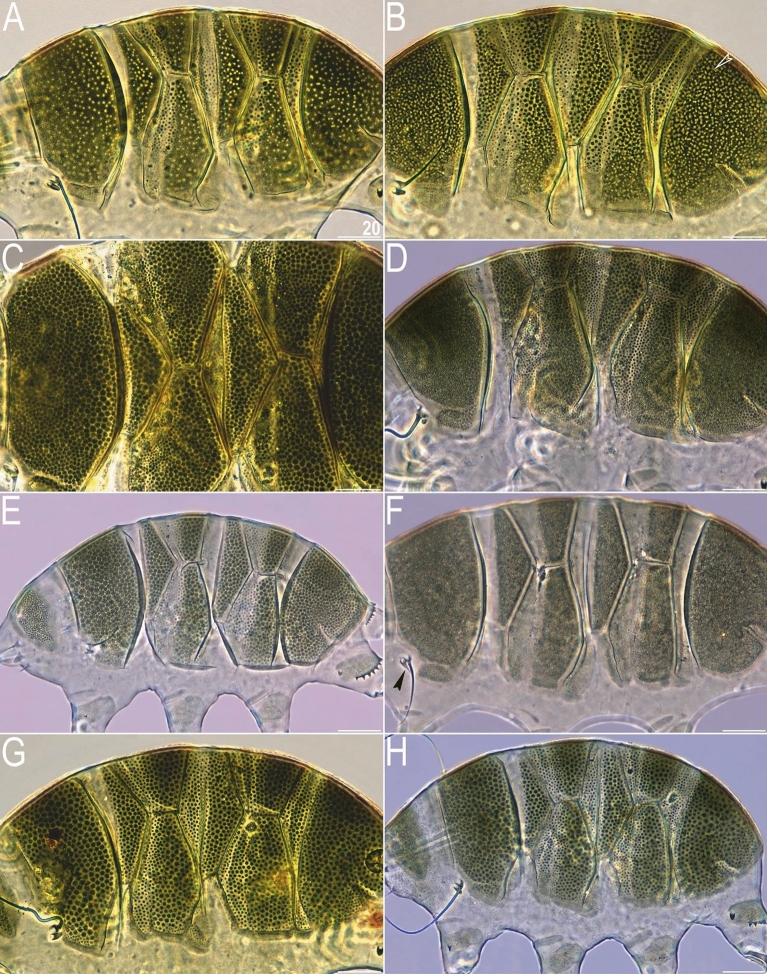
Intrageneric and intraspecific variability in *Viridiscus* (PCM): (**A**) *V.* *viridissimus* (Tennessee), the typical morph; (**B**) *V.* *viridissimus* (Tennessee), the atypical morph with well-developed epicuticular granules^16^; (**C**) *V.* *perviridis* (Madeira), the typical morph with well-developed epicuticular granules; (**D**) *V.* *perviridis* (Alabama), the atypical morph with poorly delineated epicuticular granules; (**E**) *V.* *viridianus* (Alabama), the typical morph with well-developed epicuticular granules; (**F**) *V.* *viridianus* (Alabama), the atypical morph (male) with epicuticular granules absent (arrowhead points out conoid primary clava); (**G**,**H**) *V.* *celatus* sp. nov. (Tennessee), the only morph observed. Scale bars = 20 μm.

**Figure 4:**
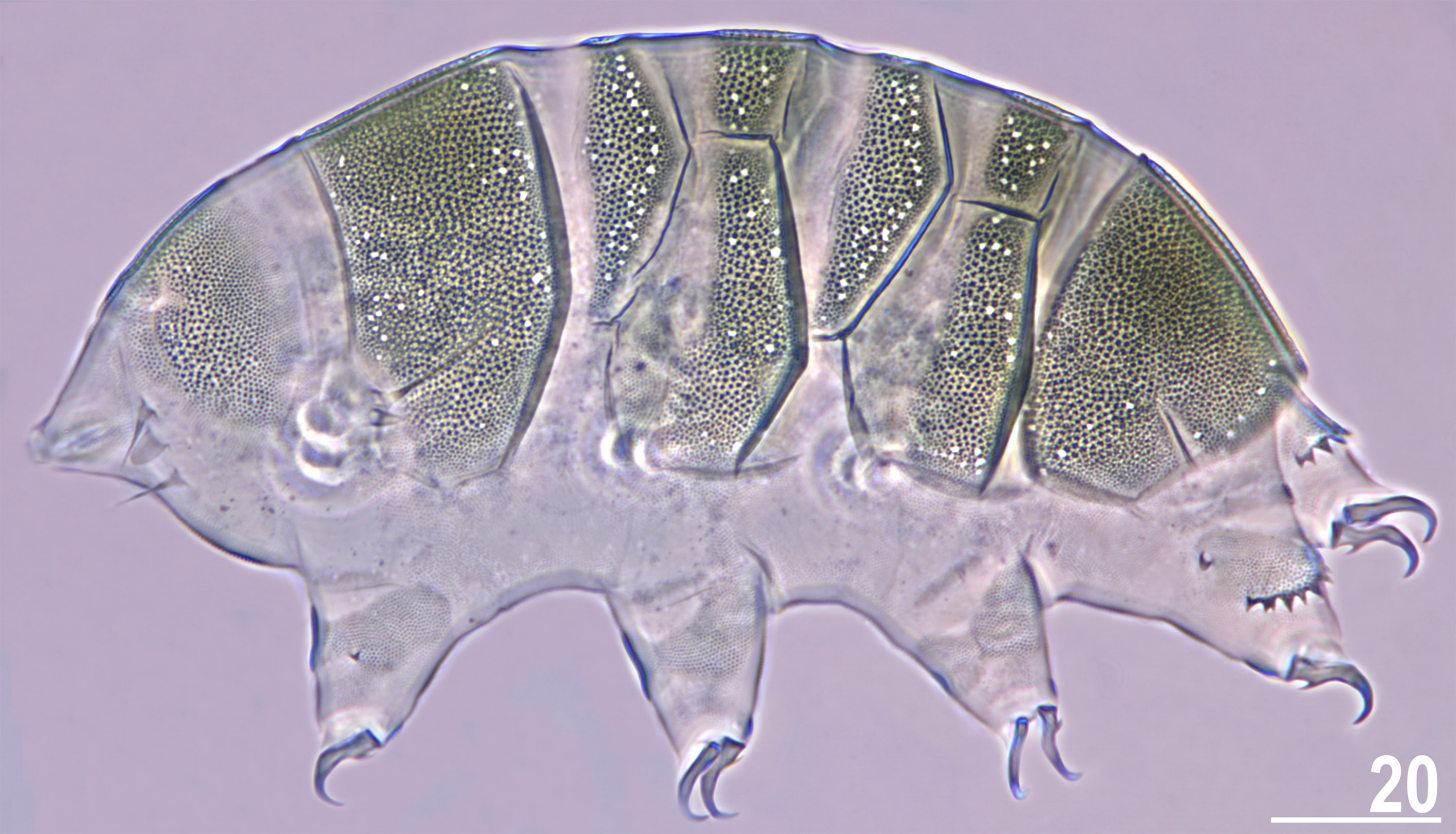
Larva of *V.* *viridianus* (PCM, dorsolateral view). Scale bar = 20 μm.

*Viridiscus* *viridissimus* exhibits considerable intra-specific variation in the morphology of dorsal armour^17^. In the typical morph (Fig. 3A), reliably recorded from the Holarctic and Oriental regions^17^, epicuticular granules are limited to the anterior portion of the second median and paired segmental plates, and to the third median plate. In contrast, the much less common morph of *V.* *viridissimus* (initially described as *V.* *miraviridis* Nelson et al., 2020^16^), so far identified only among moss samples from Tennessee, exhibits epicuticular granules in all plate portions (Fig. 3B). Having the possibility to analyse abundant populations of *V.* *perviridis* and *V.* *viridianus*, we discovered atypical morphs in these species, too. In *V.* *perviridis*, the usually well-discernible epicuticular granules (Fig. 3C) can be poorly developed (and therefore blurred with endocuticle in PCM), especially in the scapular and caudal (terminal) plates (Fig. 3D). In *V.* *viridianus*, the reduction of epicuticular granules (Fig. 3E) can be even more pronounced to the extent that granules are absent, and only the intracuticular sponge layer is identifiable in PCM (Fig. 3F). These atypical morphs were not associated with a particular life stage or sex but appeared in large monospecific populations of the analysed *Viridiscus* spp. No atypical morphs were observed in the case of *V.* *celatus* sp. nov. (Fig. 3G,H), but the available sample size was significantly smaller in the case of the new species compared to the other analysed *Viridiscus* spp. As already stated in the diagnosis of the genus^15^, all known larvae of *Viridiscus* spp., irrespectively of the adult morphotype (type I or II), possess large pores beside granules (Fig. 4). We confirmed the presence of such pores in larvae of *V.* *perviridis*, *V.* *viridianus*, and *V.* *viridissimus*, and they have been also detected in *V.* *clavispinosus*^24^. Larvae of *V.* *viridis* *s.s*. (inhabiting the Hawaiian Archipelago) have never been found^19,26^.

**Figure 5:**
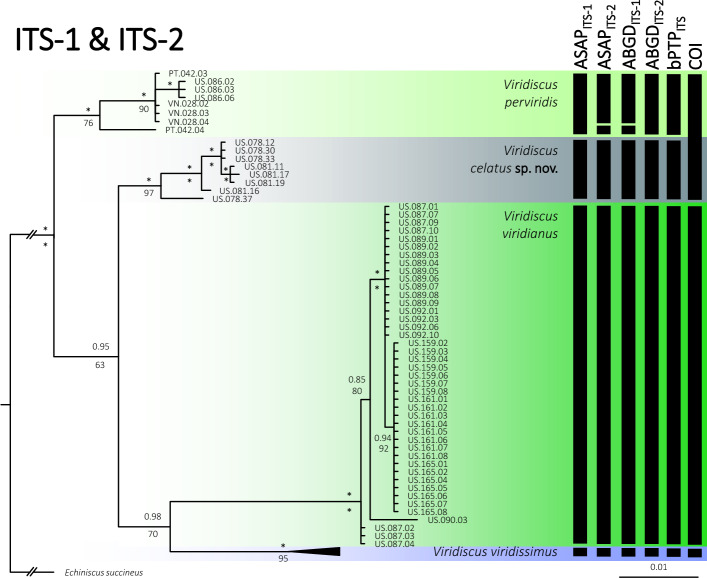
Phylogenetic relationships of the genus *Viridiscus*: Bayesian tree based on the concatenated ITS-1 + ITS-2 dataset (1058 bp); vertical bars denote different delineation methods used in the formulation of the primary molecular species hypotheses: ASAP, ABGD, and bPTP; (COI) refers to COI delimitation in all three methods). Asterisks indicate the maximal (1.00/100) posterior probability/bootstrap value. *Echiniscus* *succineus* was used as an outgroup. Scale bar represents substitutions per position.

The phylogeny fully conformed with the morphological analyses, indicating the presence of four species (Fig. 5), relationships among which were as follows: (((*V.* *perviridis* (*V.* *celatus* **sp. nov.** (*V.* *viridianus* + *V.* *viridissimus*))). *Viridiscus* *celatus* **sp. nov.** was classified in^17^ as *V*. aff. *viridianus*. Importantly, species delimitations based on COI did not distinguish between *V.* *perviridis* and *V.* *celatus* **sp. nov.**, which are clearly separated based on both ITS markers (Fig. 5) and morphology, pinpointing the questionable utility of the COI marker in tardigrade species delimitation. Given the abundant material and the proximity of the type locality in Auburn (Alabama) to the localities sampled in this study, we redescribe *V.* *viridianus* to provide a detailed insight into its intraspecific variability.

### Integrative redescription of *Viridiscus viridianus* (Pilato et al., 2007)

**Figure 6:**
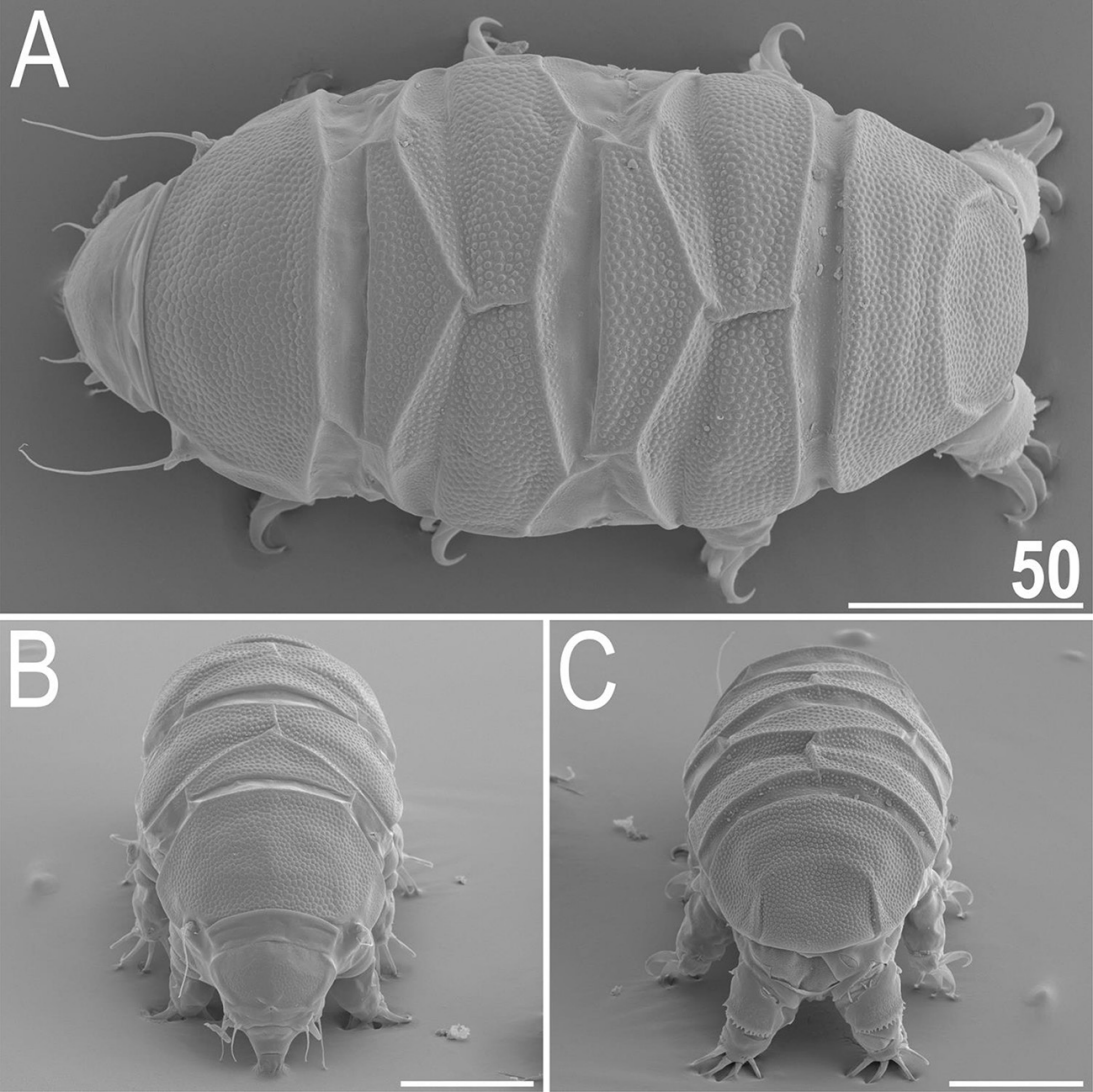
Habitus of *V.* *viridianus* (SEM): (**A**) dorsal view; (**B**) frontal view; (**C**) rear view. Scale bars = 50 μm.

**Figure 7:**
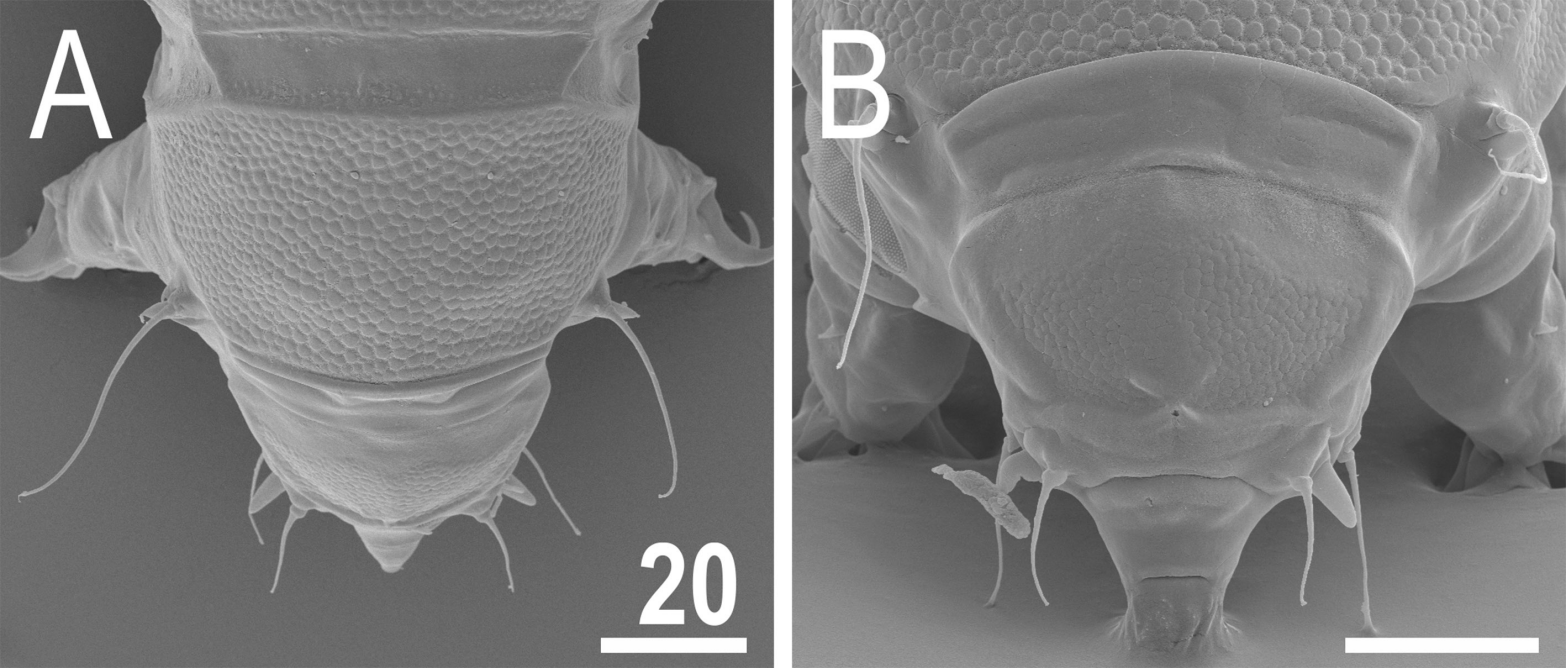
Anterior body portion of *V.* *viridianus* (SEM): (**A**) dorsal view; (**B**) head. Scale bars = 20 μm.

**Figure 8:**
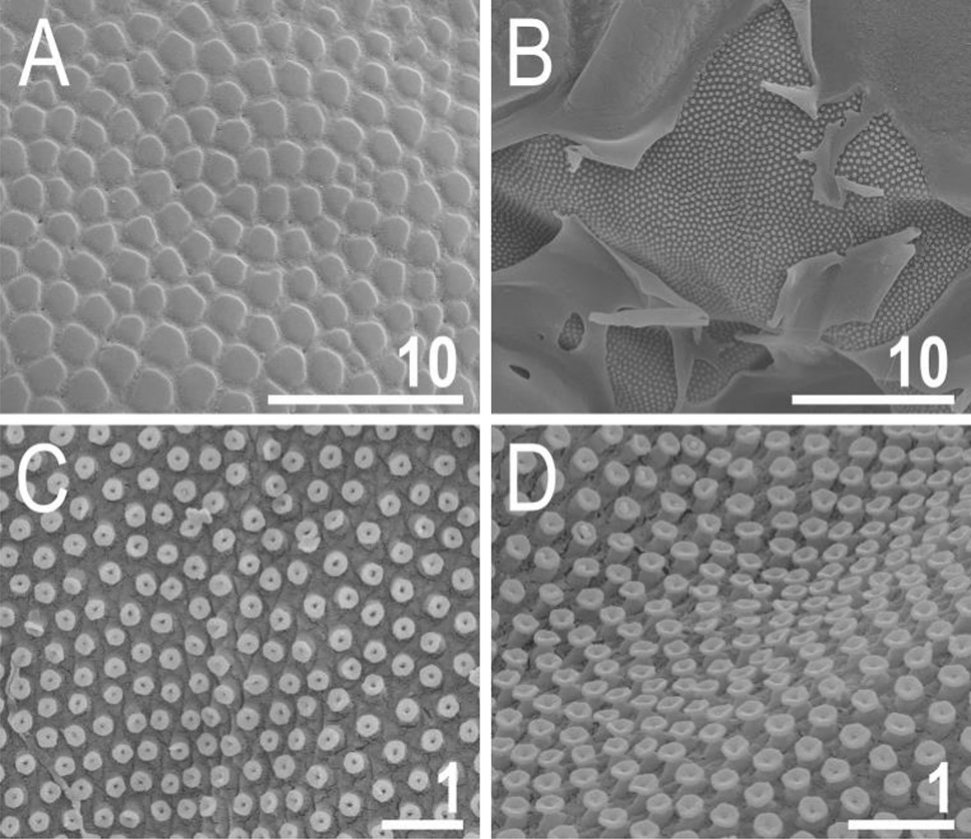
Cuticle of *V.* *viridianus* (SEM): (**A**) epicuticular granules and micropores; (**B**) lateral portion of disrupted epicuticle; (**C**,**D**) close up of intracuticular pillars. Scale bars in μm.

**Figure 9:**
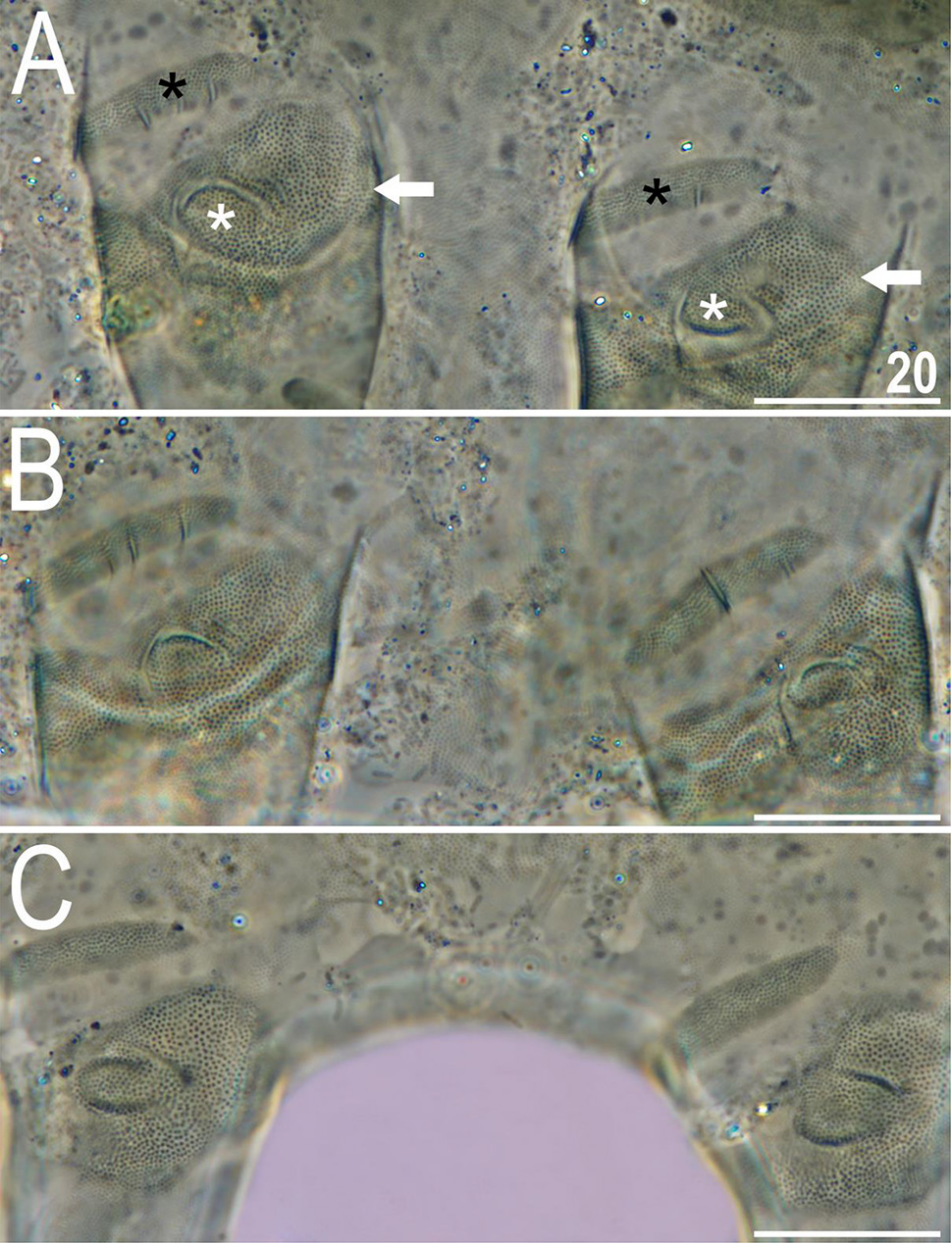
Leg morphology of *V.* *viridianus* (PCM). Arrows indicate pedal platelets in central leg portions, white asterisks indicate distinctly demarcated, central oval areas in pedal platelets, and black asterisks indicate pulvini in proximal leg portions. Scale bars = 20 μm.

**Figure 10:**
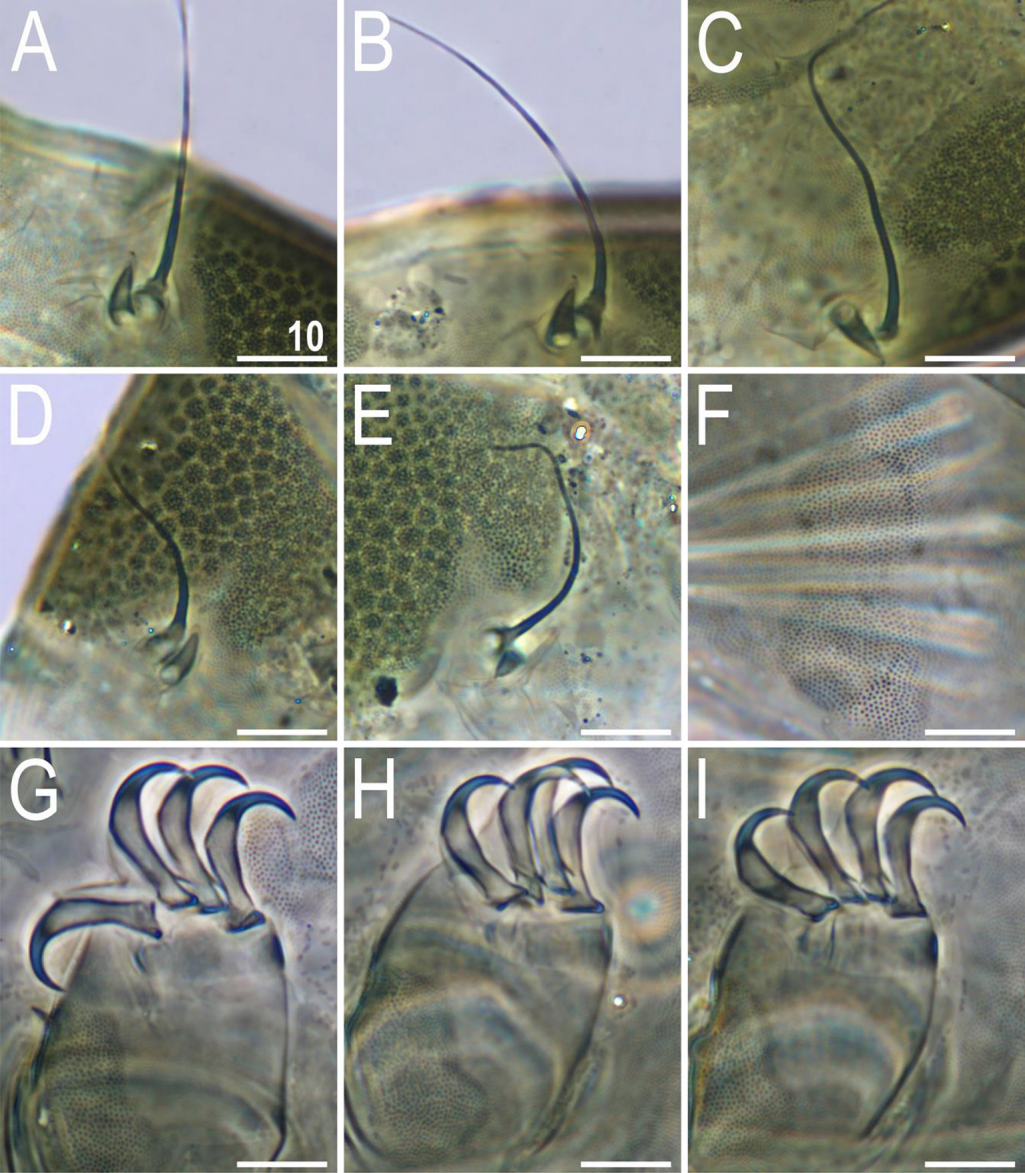
Morphological details of *V.* *viridianus* (PCM): (**A**–**C**) conoid primary clava; (**D**) dactyloid primary clava; (**E**) typical for most echiniscids, tubby *Echiniscus*-like primary clava; (**F**) subcephalic plates; (**G**) claws I; (**H**) claws II; (**I**) claws III. Scale bars = 10 μm.

**Figure 11:**
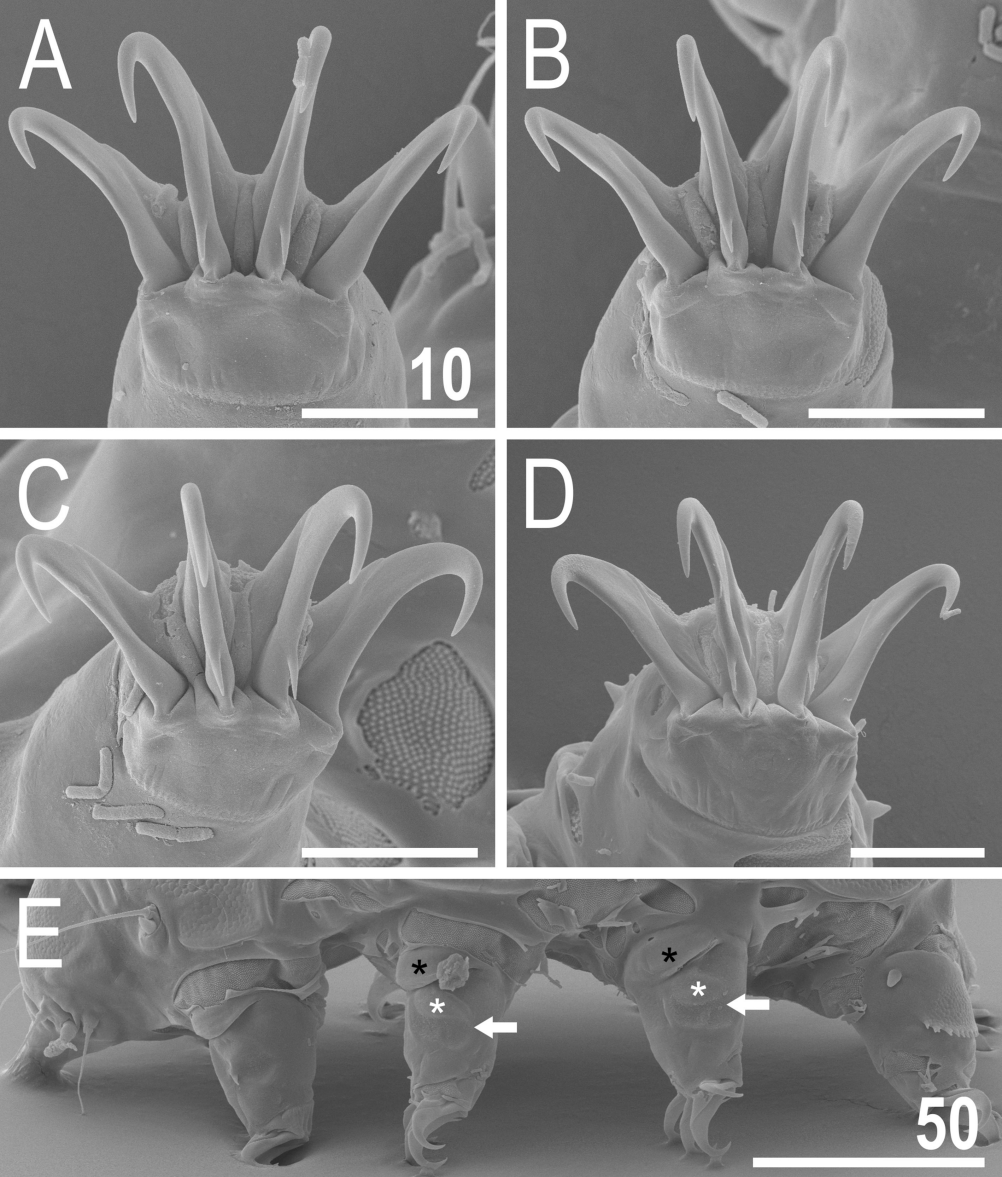
Leg structures of *V.* *viridianus* (SEM): (**A**) claws I; (**B**) claws II; (**C**) claws III; (**D**) claws IV; (**E**) leg morphology. Arrows indicate pedal platelets in central leg portions, white asterisks indicate distinctly demarcated, central oval areas in pedal platelets, and black asterisks indicate pulvini in proximal leg portions. Scale bars in μm.

(Tables 1, 2, 3, 4, Figs. 6, 7, 8, 9, 10, 11, raw morphometry in Supplementary Material 1).

**Table 1:** Measurements (in µm) of selected morphological structures of adult females of *Viridiscus* *viridianus* mounted in Hoyer’s medium. *sp* the proportion between the length of a given structure and the length of the scapular plate, ? unknown.

Character	N	Range		Mean		SD	
		µm	*sp*	µm	*sp*	µm	*sp*
Body length	15	220–326	*457–573*	280	*527*	29	*29*
Scapular plate length	15	44.3–57.9	–	53.0	*–*	3.6	*–*
Head appendage lengths							
Cirrus *internus*	14	9.0–16.7	*16.8–28.8*	12.5	*23.5*	2.0	*2.9*
Cephalic papilla	15	6.4–8.9	*12.4–16.3*	7.9	*14.9*	0.7	*1.0*
Cirrus *externus*	15	14.0–24.6	*28.2–42.5*	18.7	*35.2*	2.9	*3.9*
Clava	15	5.4–7.6	*10.3–13.3*	6.4	*12.1*	0.7	*0.9*
Cirrus *A*	15	24.5–46.1	*53.0–79.9*	37.7	*70.9*	5.9	*8.0*
Cirrus *A*/body length ratio	15	9–15%	–	13%	*–*	2%	*–*
Body appendage lengths							
Spine on leg I length	14	2.2–3.6	*4.3–6.9*	2.7	*5.1*	0.4	*0.7*
Papilla on leg IV length	15	3.0–4.6	*6.7–8.6*	3.9	*7.3*	0.4	*0.5*
Number of teeth on the collar	15	9–12	–	10.9	*–*	1.1	*–*
Claw I heights							
Branch	15	15.8–19.7	*28.5–36.6*	17.4	*32.9*	1.2	*2.1*
Spur	13	1.9–2.5	*3.3–5.2*	2.2	*4.1*	0.2	*0.5*
Spur/branch height ratio	13	10–14%	–	13%	*–*	1%	*–*
Claw II heights							
Branch	15	14.9–19.0	*30.9–33.6*	17.0	*32.0*	1.0	*0.8*
Spur	15	1.9–2.5	*3.5–5.4*	2.2	*4.1*	0.2	*0.4*
Spur/branch height ratio	15	11–16%	–	13%	*–*	1%	*–*
Claw III heights							
Branch	15	14.7–19.0	*29.8–33.2*	16.8	*31.8*	1.2	*1.1*
Spur	14	1.7–2.4	*3.2–4.7*	2.1	*3.9*	0.2	*0.4*
Spur/branch height ratio	14	10–15%	–	12%	*–*	1%	*–*
Claw IV heights							
Branch	15	17.3–20.9	*33.7–39.1*	19.3	*36.4*	1.1	*1.3*
Spur	11	2.4–3.5	*4.4–6.3*	2.9	*5.3*	0.4	*0.5*
Spur/branch height ratio	11	12–17%	*–*	15%	*–*	2%	*–*

**Table 2:** Measurements (in µm) of selected morphological structures of adult males of *Viridiscus* *viridianus* mounted in Hoyer’s medium. *sp* the proportion between the length of a given structure and the length of the scapular plate, ? unknown.

Character	N	Range		Mean		SD	
		µm	*sp*	µm	*sp*	µm	*sp*
Body length	4	248–257	*521–579*	254	*546*	4	*24*
Scapular plate length	4	42.8–48.9	–	46.6	*–*	2.6	*–*
Head appendage lengths							
Cirrus *internus*	4	11.9–13.4	*26.2–28.3*	12.7	*27.2*	0.7	*1.0*
Cephalic papilla	4	6.7–8.1	*15.7–17.1*	7.7	*16.4*	0.6	*0.6*
Cirrus *externus*	4	14.1–19.3	*32.9–40.7*	17.1	*36.5*	2.3	*3.4*
Clava	4	5.4–7.1	*12.6–15.0*	6.5	*13.9*	0.8	*1.1*
Cirrus *A*	3	25.2–37.1	*58.9–78.3*	33.1	*71.7*	6.8	*11.1*
Cirrus *A*/body length ratio	3	10–14%	–	13%	*–*	2%	*–*
Body appendage lengths							
Spine on leg I length	4	1.5–3.0	*3.5–6.3*	2.5	*5.4*	0.7	*1.3*
Papilla on leg IV length	4	3.0–4.0	*7.0–8.4*	3.7	*7.9*	0.5	*0.6*
Number of teeth on the collar	4	9–14	–	10.5	*–*	2.4	*–*
Claw I heights							
Branch	4	15.2–17.1	*33.8–35.5*	16.1	*34.6*	0.8	*0.8*
Spur	3	1.7–2.2	*3.5–5.1*	2.0	*4.4*	0.3	*0.9*
Spur/branch height ratio	3	10–14%	–	13%	*–*	2%	*–*
Claw II heights							
Branch	4	14.5–16.2	*32.1–33.9*	15.3	*32.9*	0.7	*0.8*
Spur	4	1.8–2.3	*4.2–4.9*	2.1	*4.4*	0.2	*0.3*
Spur/branch height ratio	4	12–15%	–	13%	*–*	1%	*–*
Claw III heights							
Branch	4	13.5–16.2	*31.5–33.1*	15.1	*32.4*	1.1	*0.7*
Spur	4	1.8–2.2	*3.7–4.7*	2.0	*4.3*	0.2	*0.5*
Spur/branch height ratio	4	11–15%	–	13%	*–*	2%	*–*
Claw IV heights							
Branch	4	17.3–19.4	*36.6–40.4*	18.0	*38.7*	1.0	*1.7*
Spur	3	2.7–3.1	*5.9–6.5*	2.9	*6.3*	0.2	*0.3*
Spur/branch height ratio	3	16–17%	*–*	16%	*–*	1%	*–*

**Table 3:** Measurements (in µm) of selected morphological structures of juveniles of *Viridiscus* *viridianus* mounted in Hoyer’s medium. *sp* the proportion between the length of a given structure and the length of the scapular plate, ? unknown.

Character	N	Range		Mean		SD	
		µm	*sp*	µm	*sp*	µm	*sp*
Body length	7	124–202	*419–582*	171	*503*	26	*49*
Scapular plate length	7	29.6–39.2	–	33.9	*–*	3.2	*–*
Head appendage lengths							
Cirrus *internus*	6	6.9–9.2	*20.2–25.6*	7.9	*23.2*	1.0	*2.0*
Cephalic papilla	7	5.0–6.2	*14.3–18.1*	5.5	*16.2*	0.4	*1.4*
Cirrus *externus*	7	7.8–14.4	*25.1–36.7*	10.1	*29.5*	2.2	*3.6*
Clava	6	4.1–5.3	*11.5–15.5*	4.5	*13.1*	0.4	*1.5*
Cirrus *A*	5	20.2–26.9	*58.9–72.6*	22.5	*65.0*	2.7	*5.7*
Cirrus *A*/body length ratio	5	12–17%	–	13%	*–*	2%	*–*
Body appendage lengths							
Spine on leg I length	5	1.4–1.7	*4.0–4.6*	1.5	*4.4*	0.1	*0.2*
Papilla on leg IV length	7	2.0–2.8	*6.4–8.4*	2.5	*7.3*	0.3	*0.6*
Number of teeth on the collar	6	6–11	–	8.3	*–*	1.8	*–*
Claw I heights							
Branch	7	9.4–13.2	*30.9–33.7*	10.9	*32.1*	1.3	*1.0*
Spur	7	1.4–2.0	*4.0–5.8*	1.7	*5.1*	0.2	*0.6*
Spur/branch height ratio	7	12–19%	–	16%	*–*	2%	*–*
Claw II heights							
Branch	7	9.0–13.0	*30.0–33.2*	10.5	*31.1*	1.3	*1.2*
Spur	7	1.3–1.9	*4.0–5.9*	1.7	*4.9*	0.2	*0.6*
Spur/branch height ratio	7	13–18%	–	16%	*–*	2%	*–*
Claw III heights							
Branch	7	8.4–12.2	*28.3–31.1*	10.1	*29.7*	1.3	*1.1*
Spur	6	1.4–1.8	*4.0–4.7*	1.6	*4.5*	0.2	*0.2*
Spur/branch height ratio	6	13–16%	–	15%	*–*	1%	*–*
Claw IV heights							
Branch	7	10.1–14.3	*32.4–36.8*	11.9	*34.9*	1.5	*1.7*
Spur	7	1.7–2.3	*5.2–7.1*	2.0	*6.0*	0.2	*0.7*
Spur/branch height ratio	7	14–20%	*–*	17%	*–*	2%	*–*

**Table 4:** Measurements (in µm) of selected morphological structures of larvae of *Viridiscus* *viridianus* mounted in Hoyer’s medium. *sp* the proportion between the length of a given structure and the length of the scapular plate, ? unknown.

Character	N	Range		Mean		SD	
		µm	*sp*	µm	*sp*	µm	*sp*
Body length	7	116–147	*431–535*	134	*504*	13	*44*
Scapular plate length	7	25.7–27.5	–	26.6	*–*	0.8	*–*
Head appendage lengths							
Cirrus *internus*	6	5.2–6.8	*19.0–25.1*	5.8	*21.5*	0.6	*2.1*
Cephalic papilla	6	4.4–4.9	*16.2–18.2*	4.7	*17.5*	0.2	*0.7*
Cirrus *externus*	7	6.1–8.9	*23.4–33.1*	7.4	*27.7*	0.9	*3.1*
Clava	6	3.5–4.7	*13.4–17.3*	4.2	*15.7*	0.4	*1.5*
Cirrus *A*	6	13.8–17.5	*53.5–64.6*	15.7	*58.5*	1.6	*5.0*
Cirrus *A*/body length ratio	6	10–14%	–	11%	*–*	2%	*–*
Body appendage lengths							
Spine on leg I length	7	1.3–1.5	*4.7–5.8*	1.4	*5.3*	0.1	*0.4*
Papilla on leg IV length	6	2.0–2.3	*7.3–8.6*	2.2	*8.1*	0.1	*0.5*
Number of teeth on the collar	7	6–9	–	8.0	*–*	1.2	*–*
Claw I heights							
Branch	7	9.1–10.1	*35.4–37.6*	9.7	*36.5*	0.3	*0.9*
Spur	7	1.7–2.4	*6.6–8.8*	2.0	*7.5*	0.3	*0.9*
Spur/branch height ratio	7	19–25%	–	21%	*–*	2%	*–*
Claw II heights							
Branch	7	8.7–10.0	*33.3–36.4*	9.4	*35.1*	0.5	*1.2*
Spur	7	1.7–2.2	*6.6–8.1*	1.9	*7.3*	0.2	*0.6*
Spur/branch height ratio	7	19–24%	–	21%	*–*	2%	*–*
Claw III heights							
Branch	7	8.4–9.7	*32.7–35.8*	9.1	*34.0*	0.5	*1.2*
Spur	5	1.7–2.2	*6.3–8.2*	1.9	*7.1*	0.2	*0.7*
Spur/branch height ratio	5	18–24%	–	21%	*–*	3%	*–*
Claw IV heights							
Branch	7	9.8–10.8	*37.8–40.1*	10.3	*38.7*	0.3	*0.9*
Spur	4	2.1–2.4	*7.7–8.9*	2.3	*8.5*	0.1	*0.6*
Spur/branch height ratio	4	19–23%	*–*	22%	*–*	2%	*–*

**New material examined.** Populations from Alabama and Florida, 346 specimens in total were processed for PCM, SEM and DNA analyses (Table 5); additional few hundred specimens were frozen for future analyses.

**Table 5:** List of newly found populations used in analyses. Types of analyses: (PCM) imaging and morphometry in PCM, (SEM) imaging in SEM, (DNA) DNA sequencing. Number in each analysis indicates how many specimens were utilised in a given method (*a* adults, *v* exuvia, *j* juveniles, *l* larvae).

Species	Sample code	Coordinates altitude	Locality	Sample type	Collector	Analyses		
						PCM	SEM	DNA
*Viridiscus* *celatus* sp. nov.*	US.078	36°18′N 82°22′W *ca*. 520 m asl	Tennessee, Washington County, Johnson City	Moss from a concrete cap on a brick fence post	Diane R. Nelson	1a (♀)	–	4a
	US.081	36°18′N 82°22′W *ca*. 520 m asl	Tennessee, Washington County, Johnson City	Moss from a concrete cap on a brick fence post	Diane R. Nelson	6a (4♀ + 2♂), 4j	–	4a
*Viridiscus* *perviridis*	US.086	33°12′51″N 87°34′17″W 43 m asl	Alabama, Tuscaloosa, campus of University of Alabama	Lichen** from rock boulder	Sogol Momeni	7a (♀), 2j, 1l	–	6a
*Viridiscus* *viridianus*	US.087	33°16′15″N 87°28′21″W 76 m asl	Alabama, vicinity of Lake Harris	Lichen*** from rock boulder	Sogol Momeni	28a (16♀ + 12♂), 2v	–	10a
	US.089	33°17′23″N 87°29′2″W 85 m asl	Alabama, vicinity of Lake Nicol	Lichen*** from rock boulder	Sogol Momeni	23a (20♀ + 3♂), 11j, 1l	40a	10a
	US.090	33°16′11″N 87°28′12″W 67 m asl	Alabama, vicinity of Lake Harris	Lichen*** from rock boulder	Sogol Momeni	3a (♀)	–	4a
	US.092	33°17′23″N 87°29′1″W 85 m asl	Alabama, vicinity of Lake Nicol	Lichen*** from rock boulder	Sogol Momeni	30a (27♀ + 3♂), 12j	40a	10a
	US.159	33°17′22″N 87°29′0″W 82 m asl	Alabama, vicinity of Lake Nicol	Lichen*** from rock	Sogol Momeni	12a (10♀ + 2♂), 5j, 3l	20a	8a
	US.161	33°16′15″N 87°28′20″W 76 m asl	Alabama, vicinity of Lake Harris	Moss from rock	Sogol Momeni	11a (10♀ + 1♂), 5j, 4l	20a	8a
	US.165	25°45′13″N 80°22′44″W 1 m asl	Florida, University Park	Lichen from tree	Jason Pienaar	10a (6♀ + 4♂)	10a	8a

*Viridiscus aff. *viridianus* in ^17^.

**Flavoparmelia sp.

***Flavoparmelia *baltimorensis*.

**Type locality.** North America, USA, Alabama, Auburn.

**Additional localities.** North America, USA, New Mexico^23^; the Azores, Ribeira Fria, Lages do Pico^23,29,30^; North America, USA, New Jersey^28^; Central America, the Lesser Antilles, Antigua^28^. Given the reported variability in the pattern of the dorsal armour, these additional localities should be verified.

**Etymology.** From Latin *viridianus* = greenish. An adjective in nominative singular.

**Animals.** Females (i.e., from the third instar onwards; measurements and statistics in Table 1). Body cavity with yellowish pigments (typical for most echiniscids), whereas dorsal and pedal cuticular plates light to dark green (Figs. 1C, 3E,F). Red eyes and yellow pigments present in live specimens, but dissolve after mounting in Hoyer’s medium, thus only green pigmentation persists. Body bulky (Fig. 6), with a poorly delimited cephalic region (Fig. 7). The cervical (neck) plate is well-developed, but sculptureless (Fig. 7). Weakly developed lateralmost, rectangular portions of the scapular plate with a weak sculpturing (Fig. 1C). Dorsal plate sculpturing ordinarily comprising polygonal epicuticular granules with scarce micropores, barely identifiable, even with SEM (Fig. 8A). Lateral and ventral endocuticle with intracuticular pillars, visible in PCM as minute dark dots, but identifiable in SEM only when the thin epicuticle is ruptured (Fig. 8B–D). Pillars larger and more sclerotised in proximal and central limb portions, forming longitudinal, narrow pulvini, and pedal platelets, respectively (Fig. 9). Some specimens exhibit a differently formed central pedal portion, more convex than the remainder of each platelet (Figs. 9, 11E). Areas of more sclerotised pillars always form a pair of merged subcephalic plates (Fig. 10F). Cirrus *A* short (< 20% of body length) and thin. A remarkable diversity of primary clava shapes: some specimens within a population have both clavae pointed and clearly conoid (Figs. 7A, 10A–C), some have both clavae dactyloid, i.e., elongated, but without a pointed tip (Fig. 10D), and in some individuals both clavae are tubby, i.e., of a typical, *Echiniscus*-type shape (Fig. 10E). Importantly, numerous specimens showed a mixture of these shapes, that is the clava of one specimen differed in morphology from the other one on the same specimen. Claws massive and isonych (Figs. 10G–I, 11A–D).

Males (i.e., most probably from the third instar onwards; measurements and statistics in Table 2). No detectable sexual dimorphism besides the circular gonopore.

Juveniles (i.e., the second instar; measurements and statistics in Table 3). Smaller than adults, but qualitatively like them. Gonopore absent.

Larvae (i.e., the first instar; measurements and statistics in Table 4). Body size overlaps with juveniles. Anterior portions of paired segmental plates, and median plate 2 sculptureless. Large cuticular pores in the dorsal armour. No gonopore or anus.

Eggs. Up to five orange eggs per shed exuvia, but typically fewer (see^31^).

**Remarks.** Males were present in all examined populations of the species.

### Description of *Viridiscuscelatus* sp. nov. Momeni, Gąsiorek, Nelson & Michalczyk

**Figure 12:**
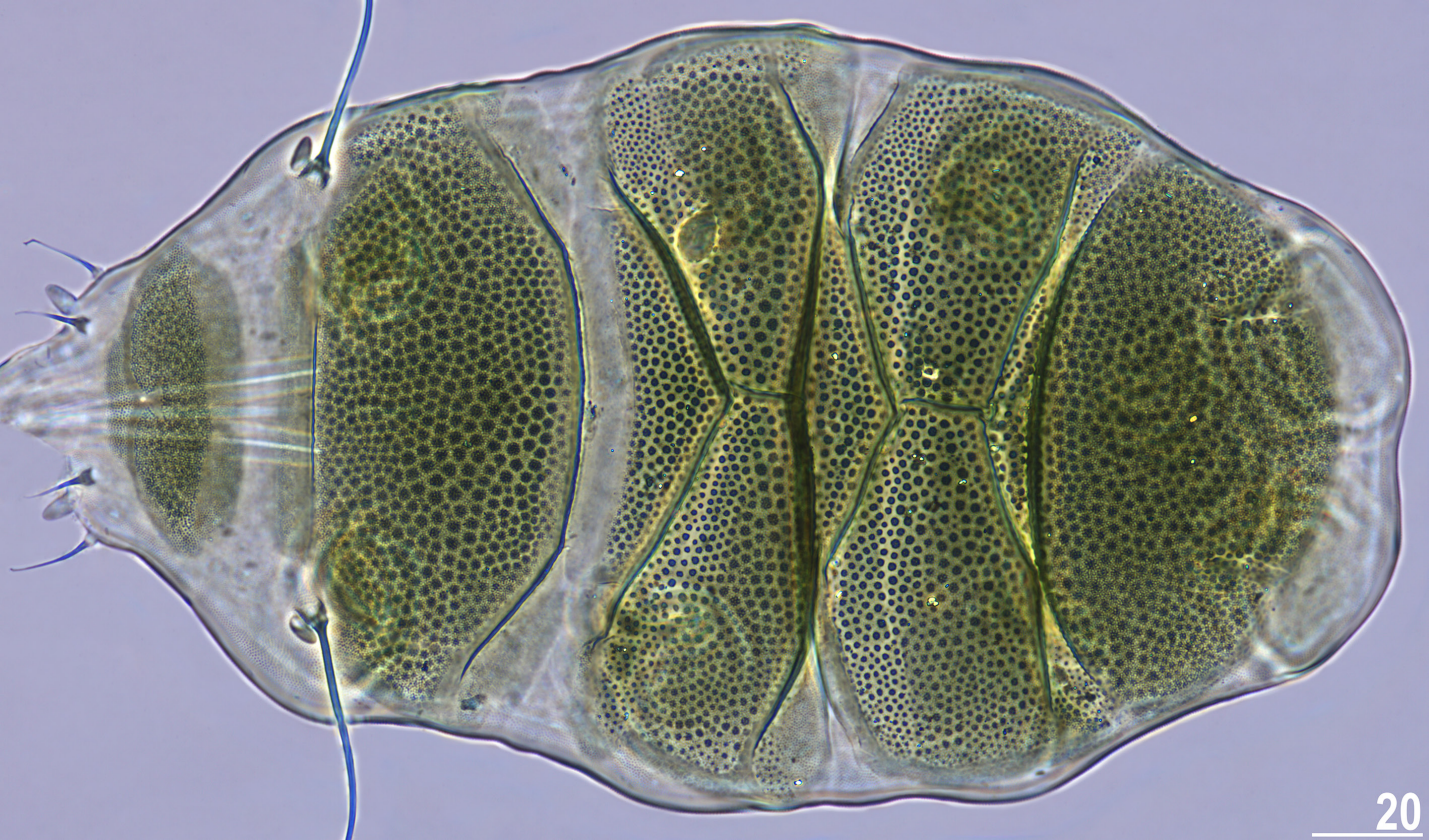
Holotype of *V.* *celatus* **sp. nov.** (PCM, female, dorsal view). Scale bar = 20 μm.

**Figure 13:**
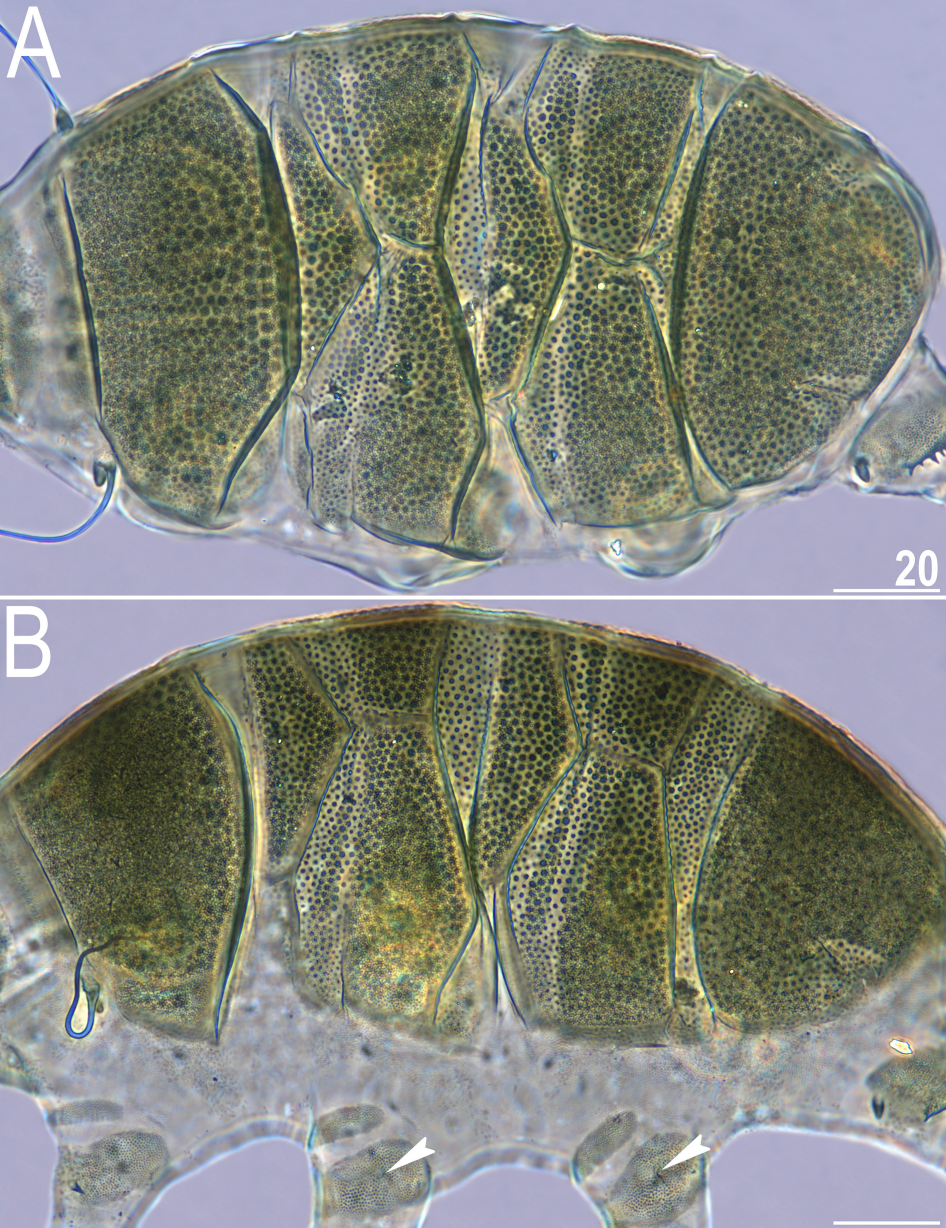
Type specimens of *V.* *celatus* **sp. nov.** (PCM): (**A**) allotype (male, dorsal view); (**B**) paratype (female, dorsolateral view). Arrowheads indicate rudimentary papillae on legs II–III. Scale bars = 20 μm.

**Figure 14:**
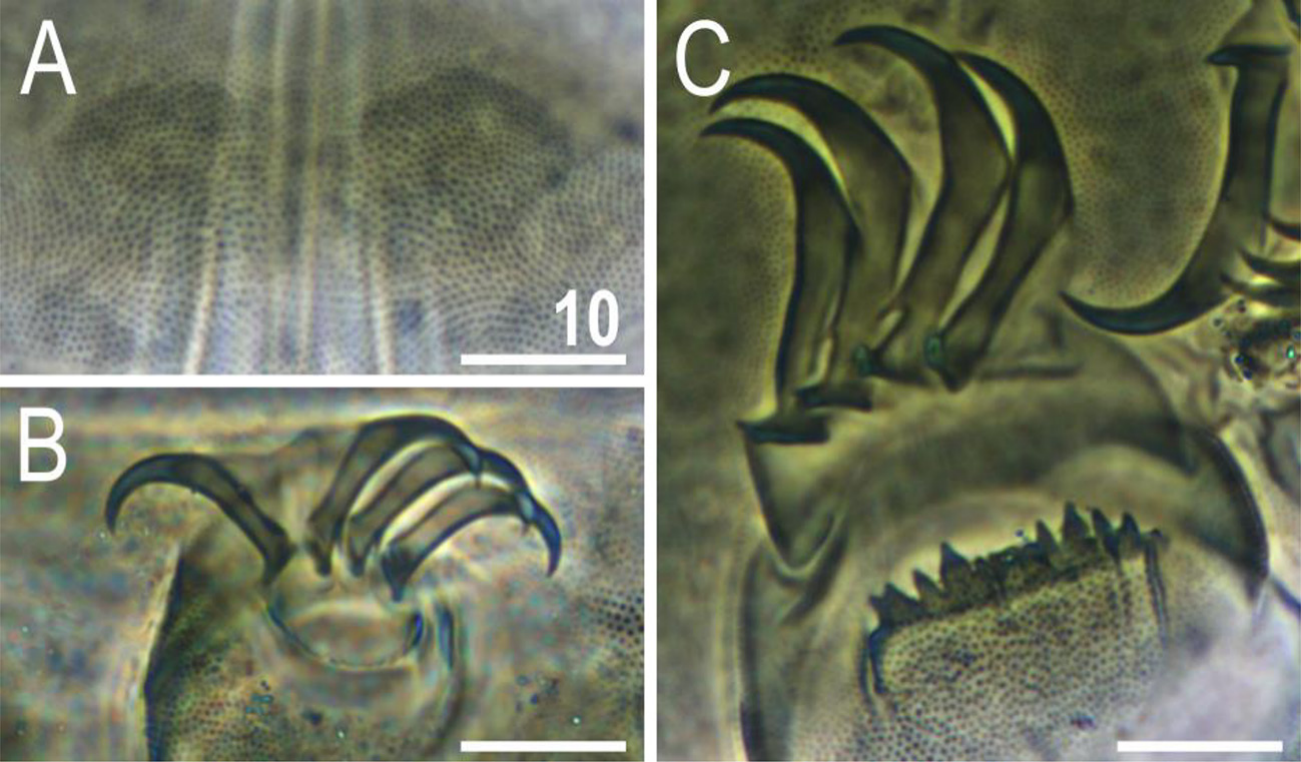
Morphological details of *V.* *celatus* **sp. nov.** (PCM): (**A**) subcephalic plates (holotype); (**B**) claws III (paratype, juvenile); (**C**) claws IV (holotype). Scale bars = 10 μm.

(Tables 6, 7, Figs. 12, 13, 14, raw morphometry in Supplementary Material 2). ZooBank registration number: urn:lsid:zoobank.org:act:7E5416A6-E49F-46C2-8BA6-3D734B2A10A4.

**Table 6:** Measurements (in µm) of selected morphological structures of adult females of *Viridiscus* *celatus* **sp. nov.** mounted in Hoyer’s medium. *sp* the proportion between the length of a given structure and the length of the scapular plate, ? unknown.

Character	N	Range		Mean		SD		Holotype	
		µm	*sp*	µm	*sp*	µm	*sp*	µm	*sp*
Body length	5	195–252	*449–533*	218	*502*	21	*32*	252	*516*
Scapular plate length	5	38.0–48.8	–	43.5	*–*	4.2	*–*	48.8	*–*
Head appendage lengths									
Cirrus *internus*	5	9.6–11.8	*23.2–27.4*	10.7	*24.7*	0.9	*2.0*	11.4	*23.4*
Cephalic papilla	5	6.2–7.1	*13.9–16.5*	6.6	*15.3*	0.3	*1.1*	7.1	*14.5*
Cirrus *externus*	5	11.7–15.2	*25.4–36.6*	13.9	*32.1*	1.5	*4.2*	15.2	*31.1*
Clava	5	5.1–6.5	*11.1–15.7*	5.9	*13.6*	0.6	*1.7*	6.3	*12.9*
Cirrus *A*	5	70.5–105.0	*152.9–243.6*	86.6	*199.8*	15.1	*32.9*	100.1	*205.1*
Cirrus *A*/body length ratio	5	34–49%	–	40%	*–*	6%	*–*	40%	*–*
Body appendage lengths									
Spine on leg I length	5	2.1–3.7	*4.9–8.7*	3.0	*6.8*	0.7	*1.5*	3.7	*7.6*
Papilla on leg IV length	5	3.8–4.7	*9.5–10.9*	4.4	*10.0*	0.4	*0.5*	4.7	*9.6*
Number of teeth on the collar	5	6–10	–	8.2	*–*	1.5	*–*	10	*–*
Claw I heights									
Branch	4	14.3–17.2	*33.0–37.6*	15.4	*35.4*	1.3	*1.9*	17.2	*35.2*
Spur	3	1.5–1.8	*3.7–4.4*	1.7	*4.0*	0.2	*0.3*	?	*?*
Spur/branch height ratio	3	10–12%	–	11%	*–*	1%	*–*	?	*–*
Claw II heights									
Branch	4	14.2–17.8	*31.2–37.8*	15.7	*35.0*	1.7	*2.9*	17.8	*36.5*
Spur	4	1.4–2.1	*3.0–4.6*	1.8	*4.0*	0.3	*0.7*	2.1	*4.3*
Spur/branch height ratio	4	10–13%	–	11%	*–*	2%	*–*	12%	*–*
Claw III heights									
Branch	5	12.3–17.0	*29.7–37.8*	14.6	*33.6*	2.0	*3.0*	17.0	*34.8*
Spur	3	1.6–1.8	*3.5–3.9*	1.7	*3.7*	0.1	*0.2*	1.8	*3.7*
Spur/branch height ratio	3	11–12%	–	11%	*–*	1%	*–*	11%	*–*
Claw IV heights									
Branch	5	15.3–21.4	*34.9–45.9*	17.7	*40.8*	2.7	*4.6*	21.4	*43.9*
Spur	4	1.6–2.3	*3.9–4.7*	1.9	*4.4*	0.3	*0.4*	2.3	*4.7*
Spur/branch height ratio	4	10–13%	*–*	11%	*–*	1%	*–*	11%	*–*

**Table 7:** Measurements (in µm) of selected morphological structures of adult males and juveniles of *Viridiscus* *celatus* **sp. nov.** mounted in Hoyer’s medium. *sp* the proportion between the length of a given structure and the length of the scapular plate, ? unknown.

Character	Allotype ♂		Paratype ♂		Juvenile 1		Juvenile 2		Juvenile 3		Juvenile 4	
	µm	*sp*	µm	*sp*	µm	*sp*	µm	*sp*	µm	*sp*	µm	*sp*
Body length	208	*470*	189	*471*	187	*475*	154	*524*	185	*532*	152	*466*
Scapular plate length	44.3	*–*	40.1	*–*	39.4	*–*	29.4	*–*	34.8	*–*	32.6	*–*
Head appendage lengths												
Cirrus *internus*	9.4	*21.2*	11.4	*28.4*	10.9	*27.7*	7.6	*25.9*	9.7	*27.9*	9.3	*28.5*
Cephalic papilla	6.3	*14.2*	5.5	*13.7*	5.9	*15.0*	5.1	*17.3*	5.5	*15.8*	4.5	*13.8*
Cirrus *externus*	10.9	*24.6*	15.4	*38.4*	12.3	*31.2*	8.2	*27.9*	11.7	*33.6*	11.8	*36.2*
Clava	5.2	*11.7*	5.0	*12.5*	5.9	*15.0*	4.3	*14.6*	5.7	*16.4*	4.8	*14.7*
Cirrus *A*	?	*?*	78.4	*195.5*	82.6	*209.6*	?	*?*	71.2	*204.6*	?	*?*
Cirrus *A*/body length ratio	?	*–*	41%	*–*	44%	*–*	?	*–*	38%	*–*	?	*–*
Body appendage lengths												
Spine on leg I length	2.8	*6.3*	2.4	*6.0*	2.7	*6.9*	2.2	*7.5*	2.6	*7.5*	1.7	*5.2*
Papilla on leg IV length	4.5	*10.2*	3.7	*9.2*	?	*?*	3.3	*11.2*	3.8	*10.9*	3.3	*10.1*
Number of teeth on the collar	9.0	*–*	11.0	*–*	?	*–*	7.0	*–*	9.0	*–*	8.0	*–*
Claw I heights												
Branch	14.0	*31.6*	15.3	*38.2*	16.5	*41.9*	9.7	*33.0*	12.9	*37.1*	11.9	*36.5*
Spur	1.6	*3.6*	2.0	*5.0*	1.9	*4.8*	1.2	*4.1*	1.6	*4.6*	1.5	*4.6*
Spur/branch height ratio	11%	*–*	13%	*–*	12%	*–*	12%	*–*	12%	*–*	13%	*–*
Claw II heights												
Branch	13.2	*29.8*	14.6	*36.4*	?	*?*	10.0	*34.0*	12.3	*35.3*	10.9	*33.4*
Spur	?	*?*	1.7	*4.2*	?	*?*	1.3	*4.4*	1.7	*4.9*	1.4	*4.3*
Spur/branch height ratio	?	*–*	12%	*–*	?	*–*	13%	*–*	14%	*–*	13%	*–*
Claw III heights												
Branch	13.4	*30.2*	14.7	*36.7*	14.3	*36.3*	9.6	*32.7*	12.1	*34.8*	10.7	*32.8*
Spur	1.5	*3.4*	1.9	*4.7*	?	*?*	1.3	*4.4*	1.6	*4.6*	1.4	*4.3*
Spur/branch height ratio	11%	*–*	13%	*–*	?	*–*	14%	*–*	13%	*–*	13%	*–*
Claw IV heights												
Branch	15.8	*35.7*	15.9	*39.7*	17.9	*45.4*	11.6	*39.5*	14.5	*41.7*	14.6	*44.8*
Spur	2.0	*4.5*	?	*?*	?	*?*	?	*?*	1.8	*5.2*	?	*?*
Spur/branch height ratio	13%	*–*	?	*–*	?	*–*	?	*–*	12%	*–*	?	*–*

**Material** **examined.** Populations from Tennessee, 19 specimens in total processed for PCM and DNA analyses (Table 5).

**Type** **locality.** 36°18′N, 82°22′W, *ca*. 520 m asl: USA, Tennessee, Washington County, Johnson City. *Grimmia* sp. mosses from a concrete cap on a brick fence post.

**Etymology.** From Latin *celatus* = hidden, concealed. The name refers to the fact that the new species was not identified as new taxon for a long time, although the locality has been extensively sampled for tardigrades (^16,27^ referred to the species as *V.* *perviridis* based on the identification by Maucci^32^, and morphological characters, including cirrus *A* length). An adjective in nominative singular.

**Type** **depositories:** Type series: holotypic female (slide US.081.03), allotypic male (slide US.081.02), and nine paratypes (slides US.078.03 and US.081.03-4), are deposited at the Faculty of Biology, Jagiellonian University (Kraków, Poland).

**Animals.** Females (i.e., from the third instar onwards; measurements and statistics in Table 6). Body medium-sized and bulky. Body cavity with yellowish pigments (typical for most echiniscids), whereas dorsal and pedal cuticular plates olive green (Figs. 1C, 3E,F). Red eyes and yellow pigments present in live specimens, but dissolve after mounting in Hoyer’s medium, thus only green pigmentation persists (Figs. 12, 13B). Except for cirrus *A*, with a tubby clava near the cirrophore (Fig. 12), other dorsal and lateral trunk appendages are absent. Cephalic appendages include internal and external peribuccal cirri with tubby cephalic papillae between them (Fig. 12). Dorsal plate sculpturing comprises large epicuticular granules (Fig. 12), which may be poorly developed in central plate portions (Fig. 13B). Sponge layer identifiable beneath granules. Granules appear more convex in anterior portions of paired segmental plates than in the remainder of the armour in PCM. Micropores not visible in PCM and their presence or absence remains to be confirmed in SEM.

All plates strongly sclerotised and with clear edges. The cephalic plate with a well-marked anterior chalice-shaped incision, the cervical plate and lateral sections of the body lack dense granulation and are covered with fine regular punctuation. The scapular plate contains three portions. Only the central part is visible in the dorsal view, and two small, weakly delineated, trapezoidal sections are present on the lateral portions of the body, with intracuticular pillars visible (Fig. 13B). The first median plate is triangular and unipartite, the second median plate is subdivided into two portions, and the anterior portion lacks the sponge layer. The third median plate is absent, but the area between the paired segmental plate II and the caudal plate is covered with large granules. Paired segmental plates I and II have two clearly delineated parts. Intersegmental plate is inserted between the posterolateral edge of the paired segmental plate I and anterior margin of paired segmental plate II. The caudal incisions are unsclerotised and weakly marked (Figs. 12, 13B).

Venter densely granulated in PCM (endocuticular pillars); a pair of subcephalic plates present (Fig. 14A). Gonopore hexapartite. Pulvini (= narrow proximal bands of intracuticular pillars) and pedal platelets (= broad central bands of pillars) are visible on all legs. Dentate collar with numerous irregular teeth (Fig. 14C). Sensory organs present on all legs: a tiny spine on leg I embedded at the edge of pedal platelet; hemispherical rudimentary papillae on legs II–III, embedded in the centre of pedal platelets (identifiable only when specimens are dorsolaterally oriented); and papilla IV on hind legs (Fig. 13B). Claws anisonych; primary spurs I–III tiny and thin, positioned slightly lower on branches compared to more massive spurs IV (Fig. 14B–C).

Males (i.e., most probably from the third instar onwards; measurements and statistics in Table 7). No sexual dimorphism observable in body size or qualitative traits (Fig. 13A). Gonopore circular.

Juveniles (i.e., the second instar; measurements and statistics in Table 7). Smaller than both females and males. Qualitatively like adults; gonopore absent.

Larvae. Not found.

Eggs. Not found.

**Remarks.** Found only in association with large populations of *V.* *viridissimus*^17^.

**Differential** **diagnosis.** The new species from Tennessee is differentiated from all *Viridiscus* spp. based on the presence of plesiomorphic papillae on legs II–III. These structures are, however, barely identifiable in specimens oriented dorsoventrally, hence we enumerate other criteria making *V.* *celatus* **sp. nov.** distinct from:

- *Viridiscus* *clavispinosus*, by the relative length of cirrus *A* (34–49% vs < 15% of the body length), and primary spurs IV less divergent from claw branches;
- *Viridiscus* *perviridis*, veritably reported from the Holarctic and Oriental regions^17^, by the length of cirrus *A* (34–49% vs typically  ≫ 50% of the body length, see the subsection below that addresses this character), the weakly developed caudal incisions (strongly sclerotised and well-marked in all syntypes of *V.* *perviridis*), and the body colour (light to olive green vs usually dark green to almost black in *V.* *perviridis*, also in mounted specimens);
- *Viridiscus* *viridianus*, reliably reported only from the USA, by the relative length of cirrus *A* (34–49% vs < 20% of the body length), the lack of pedal platelets with a distinctly formed central portion, and the more pronounced sculpturing of the anterior portions of paired segmental plates;
- *Viridiscus* *viridis*, reliably reported only from the Hawaiian Archipelago^19,26^, by the relative length of cirrus *A* (34–49% vs < 10% of the body length), and a different pattern of dorsal sculpturing (in general *V.* *viridis* has noticeably fewer epicuticular granules on all plates, see fig. 1 in^26^);
- *Viridiscus* *viridissimus*, with a likely wide distribution in the Holarctic, Oriental, and Neotropical regions^17,33^, by the absence of pores in dorsal armour, and a better developed sponge layer of cuticle.

## Discussion

### *Viridiscus viridianus* vs *Viridiscus clavispinosus*

*Viridiscus* *viridianus* was described by Pilato et al.^23^ based on populations from Alabama (the origin of the holotype, thus *locus* *typicus*), New Mexico (the Nearctic region), and the Azores (Macaronesia, the westernmost part of the Palaearctic region). A few years later, Fontoura et al.^24^ described *V.* *clavispinosus* (from the Archipelago of Cape Verde, which is also a part of Macaronesia, but located southwards of the Azores), using a seemingly sound autapomorphy, namely conoid primary clavae (with pointed apices). However, the data gathered in the present study undermine the validity of *V.* *clavispinosus*, as the shape of primary clava is evidently a variable character in some species of *Viridiscus* (Fig. 10A–E), rendering it unsuitable for species delineation. The surface between the paired segmental plate II and the caudal plate can be weakly sculptured (Figs. 1C, 3E, 6A,C) or unsculptured (Fig. 3F) in a population of one species. Contrary to what was stated in^24^, neither *V.* *viridianus* nor *V.* *clavispinosus* exhibit well-marked epicuticular granules in the cephalic and cervical plates (compare figs. 1b, e in^23^, fig. 1a in^24^, and Fig. 7 herein). In echiniscid species, the dorsal plate sculpturing is less pronounced in these two anteriormost elements of armour. The sculpturing of the less sclerotised anterior portion of median plate 2 can also be more or less developed, and sometimes absent within a single population. The remaining characters referring to the dorsal sculpturing mentioned in^24^ also are invalid in light of various atypical morphs presented for *Viridiscus* species in this paper, including *V.* *viridianus*. We refrained however, from synonymising *V.* *clavispinosus* with *V.* *viridianus* for two reasons: (1) as rightly stated by Fontoura et al.^24^, primary spurs IV of *V.* *clavispinosus* are more developed than those of *V.* *viridianus* (compare fig. 2c in^24^ and Fig. 11D herein); (2) although *V.* *viridianus* potentially has a wide circum-Atlantic distribution (whilst molecular evidence is lacking), *V.* *clavispinosus* is known from a different part of Macaronesia. Therefore, *V.* *clavispinosus* is designated as ***nomen inquirendum***, given the substantial doubts regarding its separateness from *V.* *viridianus* described above.

### Inter- vs intraspecific variability in *Viridiscus* and its consequences for echiniscid taxonomy

One of the prime tasks for taxonomists is recognising the borders of inter- and intraspecific variability in a given group of organisms (for microscopic animals, see e.g.^34–37^). In the preliminary study that integratively addressed variability of *Viridiscus*^17^, we accentuated the role of combining molecular and morphological approaches in tardigrade taxonomy, as utilising only one line of evidence either leads to taxonomic inflation (e.g., the synonymy of *V.* *miraviridis*^16,17^) or deflation (e.g., the *Milnesium* case^38^). We have already underscored high variability of characters previously deemed as universally stable at the species level in the Echiniscidae (cirrus *A* length, details of cuticular ornamentation) in *Echiniscus* and *Nebularmis* spp.^39,40^. This study adds further observations on atypical morphs within *Viridiscus*, which show disparities with typical morphs of a given species that are larger than interspecific variability in multiple new characters, such as the general phenotype of the dorsal sculpturing, the shape of primary clava, and morphology of pedal platelets. It is gradually becoming obvious that even within such a morphologically coherent family as Echiniscidae^41^, phenotypic plasticity of diagnostic characters varies between lineages at various taxonomic levels (genus, species, etc.), as it happens with the shape of primary clavae in *Viridiscus*, which is labile in contrast to other known echiniscid genera. Consequently, conservative characters in one genus should not simply be assumed to be conservative in other taxa. In parallel, analogous variability in characters such as the shape and presence of spine I and dorsal sculpturing, has been also reported recently in *Claxtonia* spp.^42^.

On the other hand, genetic data, when taken in isolation, can also be deceiving, and this pertains specifically to the COI marker, often uncritically utilised and termed as a universal tardigrade barcode as (I) it is often difficult to amplify, and (II) may fail in delimiting species (Fig. 5;^43^). Multiple markers are advisable^44^, and both ITS markers are good predictors of intra- and interspecific differences, as they are congruent with a spectrum of morphological variability analysed on many animals. Altogether, this reinforces the necessity of the integrative approach in tardigrade studies^17^.

In the light of our findings, some generalisations can be made regarding the diversity and classification of *Viridiscus*:

1. The green body colour is a result of dorsal cuticular plate pigmentation, but orange and yellow (probably carotenoid-derived pigments), are present in all *Viridiscus* spp., as in most other echiniscids. Thus, two characteristics of the dorsal armour, the presence of the endocuticular sponge layer and green pigmentation, are advanced characters.
2. Although more variable than previously reported, the relative length of cirrus *A* with respect to the body length (*bo*) serves as a good criterion in morphological species delineation. The extreme elongation of cirrus *A* characterises *V.* *perviridis* (most often  ≫ 50% of *bo*), followed by cirri *A* longer than the echiniscid average (30–50% of *bo*) in *V.* *celatus* **sp. nov.** and *V.* *viridissimus*, and cirri *A* that can be classified as short (< 20% of *bo*) in *V.* *clavispinosus* **nom. inq.**, *V.* *viridianus*, and *V.* *viridis*^23^.
3. Atypical morphs are probably present in populations of all *Viridiscus* spp. We recorded them in *V.* *perviridis*, *V.* *viridianus* (this study), and *V.* *viridissimus*^17^. This means that morphology alone does not offer a fully credible dataset for establishing new species.
4. The description of *V.* *viridis* by Pilato et al.^23^ was based on only two individuals and likely does not reflect the spectrum of morphological variability of this species, but plainly indicates the specific nature of its dorsal sculpturing. The sculpturing embraces particularly widely spaced epicuticular granules, making it dissimilar to all other *Viridiscus* spp. Unless integratively redescribed from the Hawaiian Islands and later verified from other regions of the globe, it should be temporarily considered an endemic of this archipelago.

## Conclusions

The extent of intraspecific morphological variability within *Viridiscus* is considerable and exceeds the usual variation encountered within echiniscid populations. Atypical morphs of *Viridiscus* species are not linked with sexual dimorphism, phenology, or ontogeny, and seem to represent idiopathic deviations from the most common morphs. *Viridiscus* *celatus* **sp. nov.**, found only in association with large populations of *V.* *viridissimus*, is described integratively based on genetics and morphology. In addition, the validity of *V.* *clavispinosus* is questioned, and its synonymy with *V.* *viridianus* is implied. Males were reported in populations of *Viridiscus* *celatus* **sp. nov.**, *V.* *viridianus*, and *V.* *viridissimus* so far.

## Materials and methods

### Sample collection and processing

**Figure 15:**
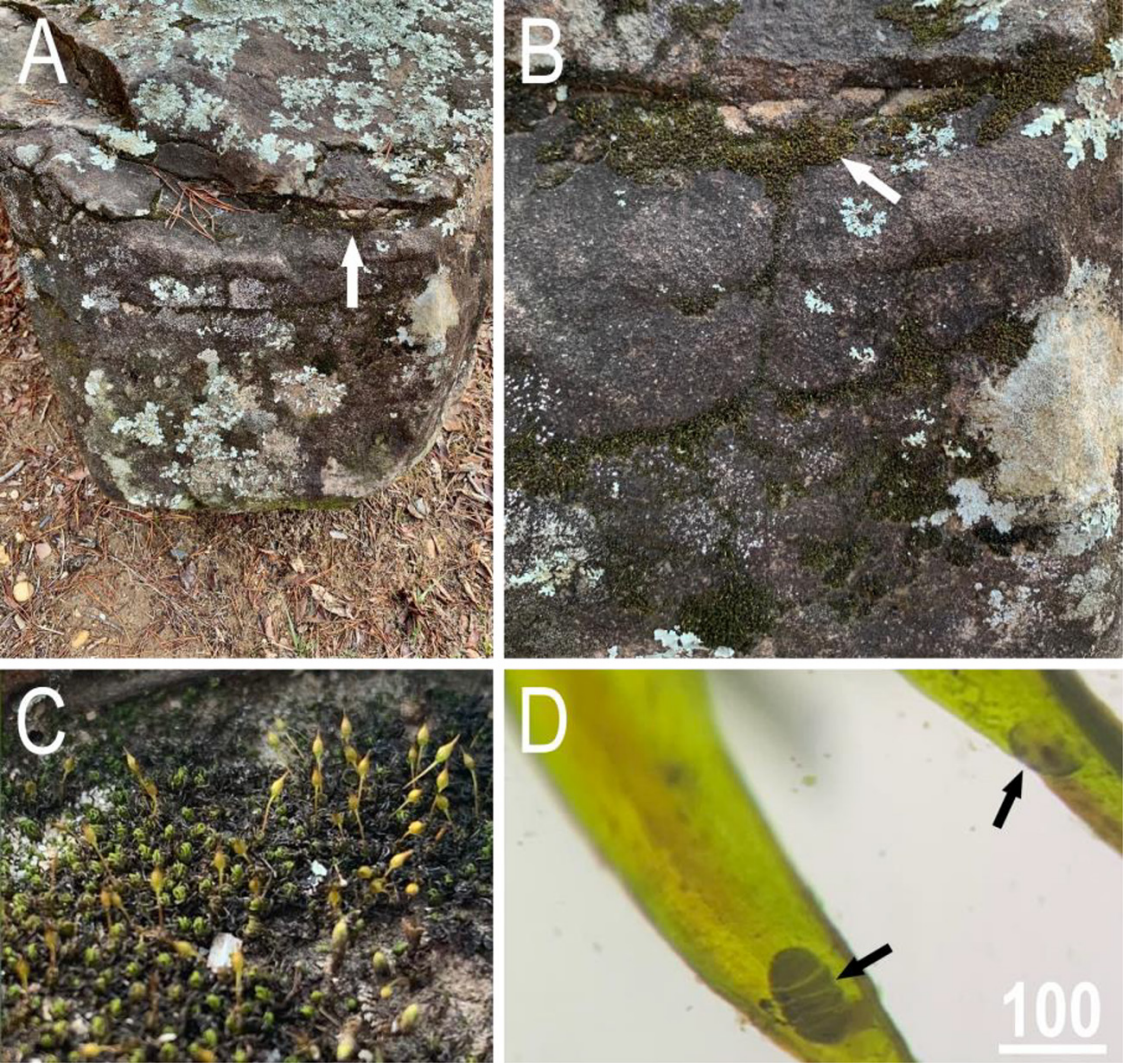
Habitats harbouring *Viridiscus* populations in *Andreaea* mosses growing on boulders by Lake Harris, Tuscaloosa, Alabama: (**A**) an overall view of the rock substrate; (**B**) close up of the moss growing on the vertical surfaces of the boulder (arrow indicates the moss matt); (**C**) close up of the pleurocarpous moss and leaf structure when dry; (**D**) moss leaves seen under a stereomicroscope (arrows indicate animals on moss leaves in the tun state).

Previous sampling experience^31,45^ showed that *Viridiscus* species are often found in mosses and lichens growing on rapidly draining vertical surfaces (see e.g.^46^) such as the sides of rocks (Fig. 15), gravestones, and tree trunks in human-altered environment. Therefore, moss and lichen samples were collected from several localities in Alabama, Florida, and Tennessee from February 2020 to May 2022 (Table 5). All samples were stored in acid-free paper envelopes and allowed to dry overnight in an air-conditioned room at 25 °C and 50% relative humidity. The air-dried samples were suspended over deionised water using the custom-built Baermann pan described by Davison^47^ for at least 12 h to separate the motile meiofauna from the moss and lichen particles. The deionised water was then filtered through 25 µm mesh and the sieve was rinsed into 30 mm Petri dishes with locally collected rainwater to search for tardigrades. The air-dried lichens were soaked in deionised water for at least 12 h, active tardigrades were extracted with an Irwin loop and transferred into 30 mm Petri dishes containing rainwater.

### Microscopy and imaging

Individual tardigrades from the samples were visually inspected for diagnostic traits using an Olympus BX53 phase contrast microscope (PCM) associated with an Olympus DP74 digital camera. All specimens were divided into morphological and molecular analyses (details in Table 5). The excess of individuals was frozen to store for future analyses. For morphology and morphometry, individuals were permanently mounted on microscope slides in Hoyer’s medium. Dried slides were sealed with nail polish and examined under the Olympus BX53 PCM. Specimens for imaging in the SEM were CO_2_ critical point-dried, coated with gold and examined in the Versa 3D DualBeam SEM at the ATOMIN facility of the Jagiellonian University. All figures were assembled in Corel Photo-Paint X8. For deep structures that could not be fully focused on a single PCM photograph, a series of images was taken every *ca*. 0.1 mm of vertical focusing and then assembled manually in Corel Photo-Paint into a single deep-focus image.

### Morphometry

All individuals of the new species from Tennessee and selected individuals of *V.* *viridianus* from Alabama were chosen for morphometry. The measurements are in micrometres (µm), and only undamaged structures with the appropriate orientation were used. The body length measurement is from the anterior to the posterior ends of the body, excluding the hind limbs. The *bo%* was calculated for cirrus *A*, which is the ratio of cirrus *A* length to the body, and *sp%* is the ratio of the length of the given structure to the length of the scapular plate^48^. Morphometric data were handled using the Echiniscoidea ver. 1.4 template available from the Tardigrada Register, http://www.tardigrada.net/register^49^.

### Comparative material

The morphology of *Viridiscus* spp. was compared with paratypes of *V.* *viridianus* and syntypes of *V.* *perviridis* (deposited in the Civic Museum of Natural History in Verona and University of Modena and Reggio Emilia, Italy), and original descriptions or redescriptions of species^17,23–26^. Slides of *V.* *viridissimus* from Tennessee, including the “*miraviridis* morphotype” (^16^, synonymised with *V.* *viridissimus* by Gąsiorek et al.^17^), were also examined.

### Genotyping

A Chelex^®^ 100 resin (Bio-Rad) extraction method was used for DNA extraction^50,51^. Hologenophores were recovered after the extraction and mounted on permanent slides in Hoyer’s medium in most cases; in other cases, paragenophores were preserved^52^; all are deposited in the Faculty of Biology, Jagiellonian University in Kraków. Five DNA fragments (nuclear markers: 18S rRNA, 28S rRNA, ITS-1, ITS-2; mitochondrial marker: COI) were amplified and sequenced according to the protocols described in^51^; primers and original references for specific PCR programmes are listed in Supplementary Material 3. GenBank accession numbers for the species used in calculating phylogenies and the genetic framework (mainly based on the dataset from Gąsiorek et al.^17^) are provided in Table 8. ITS and COI sequences were separately aligned with sequences from *Echiniscus* *succineus* Gąsiorek & Vončina, 2019^53^ as an outgroup using the ClustalW Multiple Alignment tool^54^ implemented in BioEdit ver. 7.2.5^55^. The remaining gaps were left intact in ITS alignments. The 18S and 28S rRNA gene fragments were not used for the species delimitation purposes but are provided for future broader-scale phylogenies.

**Table 8:** GenBank accession numbers for the sequences analysed in this work. New sequences in bold.

Species	Population	18S rRNA	28S rRNA	ITS-1	ITS-2	COI
*Viridiscus* *celatus* sp. nov.*	US.078	MZ868197	OK094230	OK094211–4	OK094173–6	MZ852064–6
	US.081	MZ868198	OK094231	OK094215–8	OK094177–80	MZ852067–9
*Viridiscus* *perviridis*	PT.042	MK529696	MK529726–7	OK094219–20	OK094181–2	–
	US.086	**OR519990–2**	**OR520001–3**	–	**OR520055–7**	**OR502563–5**
	VN.028	MZ868199	OK094232	OK094221–3	OK094183–5	–
*Viridiscus* *viridianus*	US.087	**OR519993–4**	**OR520004–5**	**OR520012–8**	**OR520058–64**	**OR502566–72**
	US.089	–	–	**OR520019–27**	**OR520065–73**	**OR502573–81**
	US.090	–	–	**OR520028**	**OR520074**	**OR502582**
	US.092	–	–	**OR520029–32**	**OR520075–8**	**OR502583–6**
	US.159	**OR519995–6**	**OR520006–7**	**OR520033–9**	**OR520079–85**	**OR502587–93**
	US.161	**OR519997–8**	**OR520008–9**	**OR520040–7**	**OR520086–93**	**OR502594–601**
	US.165	**OR519999–20000**	**OR520010–1**	**OR520048–54**	**OR520094–100**	**OR502602–8**
*Viridiscus* *viridissimus*	US.078	MZ868191–3	OK094224, 6–7	OK094186–90, 207–208	OK094148–52, 69–70	MZ852046–9, 62–3
	US.081	MZ868194	OK094225	OK094191–206	OK094153–68	MZ852050–61
	VN.028	MZ868195–6	OK094228–9	OK094209–10	OK094171–2	–
*Echiniscus* *succineus*	MG.005	MK675903	MK675914	MT374198	MK675925	MK649675

*Viridiscus aff. *viridianus* in^17^.

### Phylogeny

The sequences of the ITS fragments were concatenated to generate a matrix of 1058 bp in SequenceMatrix^56^. Using PartitionFinder version 2.1.1^57^ with applied Bayesian Information Criterion (BIC) and the greedy algorithm^58^, the best substitution model (the lowest BIC) and partitioning scheme were chosen for posterior phylogenetic analysis. As the best-fit partitioning scheme, PartitionFinder indicated one partition with the best-fitted model GTR + G. Bayesian inference (BI) marginal posterior probabilities were calculated using MrBayes v.3.2^59^. Random starting trees were used, and the analysis was run for ten million generations, sampling the Markov chain every 1000 generations. An average standard deviation of split frequencies of < 0.01 was used as a guide to ensure that the two independent analyses had converged. Tracer v1.3^60^ was then used to ensure Markov chains had reached stationarity and to determine the correct ‘burn-in’ for the analysis, in this case the first 10% of generations. The Effective Sample Size values were greater than 200, and the consensus tree was obtained after summarising the resulting topologies and discarding the ‘burn-in’.

ModelFinder^61^ was used to choose the best-fit models for two partitions in Maximum Likelihood (ML): TIM3e + G4 (ITS-1) and K2P + G4 (ITS-2), chosen according to the BIC. W-IQ-TREE was used for ML reconstruction^62,63^. One thousand ultrafast bootstrap (UFBoot) replicates were applied to provide support values for branches^64^.

Both ITS and COI alignments were uploaded separately to the Assemble Species by Automatic Partitioning (ASAP) webpage^65^, and automatic barcode gap discovery (ABGD) web^66^ to obtain six independent marker-based primary species hypotheses using uncorrected pairwise distances. The partitions with the lowest ASAP and ABGD scores and *p* values < 0.05 were chosen as the best-fit hypotheses. Finally, Bayesian Poisson tree processes (bPTP^67^) were applied to the ML phylogenetic trees run on three markers separately in W-IQ-TREE (COI best-fitted model: HKY + F + G4). The calculations were conducted with 100,000 MCMC generations, thinning the set to 100, with 10% burn-in, and with searches for maximum likelihood and Bayesian solutions. All final consensus trees were visualised by FigTree v.1.4.3 available from https://tree.bio.ed.ac.uk/software/figtree.

### Supplementary Information

Supplementary Information 1.

Supplementary Information 2.

Supplementary Information 3.

## Data Availability

Data generated or analysed during this study are included in the published article and its supplementary information files. All sequences are deposited in GenBank. The publication was registered in ZooBank: urn:lsid:zoobank.org:pub:E2AA1924-C159-460B-9C8B-F23D951A5FA3.
