# Supplementary material for: Green armoured tardigrades (Echiniscidae: Viridiscus), including a new species from the Southern Nearctic, exemplify problems with tardigrade variability research
Source: Sci Rep. 2023 Sep 28;13:16329. doi: 10.1038/s41598-023-40609-4 (PMC10539286; doi:10.1038/s41598-023-40609-4)
Supplement: Supplementary file 3 — Supplementary Information 3. [file 41598_2023_40609_MOESM3_ESM.docx]

**SM.3.** Primers and references for specific protocols for amplification of the five DNA fragments sequenced in the study.

| **DNA fragment** | **Primer name** | **Primer direction** | **Primer sequence (5’-3’)** | **Primer source** | **PCR programme*** |
| --- | --- | --- | --- | --- | --- |
| **18S rRNA** | 18S_Tar_Ff1 | forward | AGGCGAAACCGCGAATGGCTC | Stec et al. (2017) | Zeller (2010) |
|  | 18S_Tar_Rr2 | reverse | CTGATCGCCTTCGAACCTCTAACTTTCG | Gąsiorek et al. (2017) |  |
| **28S rRNA** | 28S_Eutar_F | forward | ACCCGCTGAACTTAAGCATAT | Gąsiorek et al. (2018) | Mironov et al.  (2012) |
|  | 28SR0990 | reverse | CCTTGGTCCGTGTTTCAAGAC | Mironov et al.  (2012) |  |
| **ITS-1** | ITS1_Echi_F | forward | CCGTCGCTACTACCGATTGG | Gąsiorek et al. (2019) | Wełnicz et al. (2011) |
|  | ITS1_Echi_R | reverse | GTTCAGAAAACCCTGCAATTCACG |  |  |
| **ITS-2** | ITS3 | forward | GCATCGATGAAGAACGCAGC | White et al. (1990) |  |
|  | ITS4 | reverse | TCCTCCGCTTATTGATATGC |  |  |
| **COI** | bcdF01 | forward | CATTTTCHACTAAYCATAARGATATTGG | Dabert et al. (2008) |  |
|  | bcdR04 | reverse | TATAAACYTCDGGATGNCCAAAAAA |  |  |

* – All PCR programmes are also provided in Stec et al. (2020).

Dabert J., Ehrnsberger R. & Dabert M. (2008) *Glaucalges tytonis* sp. nov. (Analgoidea: Xolalgidae) from the barn owl *Tyto alba* (Strigiformes: Tytonidae): compiling morphology with DNA barcode data for taxa descriptions in mites (Acari). Zootaxa 1719: 41–52. <https://doi.org/10.11646/zootaxa.1719.1.2>

Gąsiorek P., Jackson K.J., Meyer H.A., Zając K., Nelson D.R., Kristensen R.M. & Michalczyk Ł. (2019) *Echiniscus virginicus* complex: the first case of pseudocryptic allopatry and pantropical distribution in tardigrades. Biological Journal of the Linnean Society 128: 789–805. <https://doi.org/10.1093/biolinnean/blz147>

Gąsiorek P., Stec D., Morek W. & Michalczyk Ł. (2017) An integrative redescription of *Echiniscus testudo* (Doyère, 1840), the nominal taxon for the class Heterotardigrada (Ecdysozoa: Panarthropoda: Tardigrada). Zoologischer Anzeiger 270: 107–122. <https://doi.org/10.1016/j.jcz.2017.09.006>

Gąsiorek P., Stec D., Zawierucha K., Kristensen R.M. & Michalczyk Ł. (2018) Revision of *Testechiniscus* Kristensen, 1987 (Tardigrada: Heterotardigrada: Echiniscidae) refutes the polar–temperate distribution of the genus. Zootaxa 4472: 261–297. <https://doi.org/10.11646/zootaxa.4472.2.3>

Mironov S.V., Dabert J. & Dabert M. (2012) A new feather mite species of the genus *Proctophyllodes* Robin, 1877 (Astigmata: Proctophyllodidae) from the long-tailed tit *Aegithalos caudatus* (Passeriformes: Aegithalidae): morphological description with DNA barcode data. Zootaxa 3253: 54–61. <https://doi.org/10.11646/zootaxa.3253.1.2>

Stec D., Kristensen R.M. & Michalczyk Ł. (2020) An integrative description of *Minibiotus ioculator* sp. nov. from the Republic of South Africa with notes on *Minibiotus pentannulatus* Londoño et al., 2017 (Tardigrada: Macrobiotidae). Zoologischer Anzeiger 286: 117–134. <https://doi.org/10.1016/j.jcz.2020.03.007>

Stec D., Zawierucha K. & Michalczyk Ł. (2017) An integrative description of *Ramazzottius subanomalus* (Biserov, 1985) (Tardigrada) from Poland. Zootaxa 4300: 403–420. <https://doi.org/10.11646/zootaxa.4300.3.4>

Wełnicz W., Grohme M.A., Kaczmarek Ł., Schill R.O. & Frohme M. (2011) ITS-2 and18S rRNA data from *Macrobiotus polonicus* and *Milnesium tardigradum* (Eutardigrada, Tardigrada). Journal of Zoological Systematics and Evolutionary Research 49: 34–39. <https://doi.org/10.1111/j.1439-0469.2010.00595.x>

White T.J., Bruns T., Lee S. & Taylor J. (1990) PCR protocols: a guide to methods and application. San Diego: Academic Press, 315–322.

Zeller C. (2010) Untersuchung der Phylogenie von Tardigraden anhand der Genabschnitte 18S rDNA und Cytochrom c Oxidase Untereinheit 1 (COX I). MScThesis, Technische Hochschule Wildau, Wildau.
